# Mapping of plant–fungal interactions on agriculture perception: a bibliometric analysis and systematic review

**DOI:** 10.3389/fmicb.2025.1612428

**Published:** 2025-10-10

**Authors:** Suhail Asad, Mei Chen, Alviti Kankanamalage Hasith Priyashantha, Peng Gu, Jie Liu, Zhiguo Shan, Saowaluck Tibpromma, Chen Niu, Masood Qadir, Muhammad Akhtar, Xurundong Kan, Yiren Xu, Zaiqiong Liu, Samantha C. Karunarathna, Jianqiang Zhang

**Affiliations:** ^1^School of Tea and Coffee, Pu’er University, Pu’er, China; ^2^Yunnan International Union Laboratory for Quality Monitoring and Evaluation of Agricultural Products in China and Malaysia, Pu’er University, Pu’er, China; ^3^Department of Biology, Faculty of Science, Chiang Mai University, Chiang Mai, Thailand; ^4^School of Biology and Chemistry, Pu’er University, Pu’er, China; ^5^Center for Yunnan Plateau Biological Resources Protection and Utilization & Yunnan International Joint Laboratory of Fungal Sustainable Utilization in South and Southeast Asia, College of Biology and Food Engineering, Qujing Normal University, Qujing, China; ^6^Spice and Beverage Research Institute, Chinese Academy of Tropical Agriculture Sciences, Pu’er, China; ^7^Department of Plant Breeding and Genetics, Ghazi University, Dera Ghazi Khan, Pakistan; ^8^Key Laboratory of Crop Physiology and Ecology, Institute of Crop Sciences, Chinese Academy of Agricultural Sciences, Ministry of Agriculture and Rural Affairs of China, Beijing, China

**Keywords:** co-infection, endophytic fungi, gene expression, mycorrhiza, sustainable agriculture, synergistic effect

## Abstract

In nature, plants are always destined to interact with fungi. Thus, plant–fungal interactions are one of the unavoidable fields of study, particularly for agronomists. Fungi interact with plants in different lifestyles, pathogenic and symbiotic. Pathogenic relationships have adverse effects, causing devastating diseases in plants, while symbiotic interactions provide numerous benefits, promoting the growth and development of plants. The intricate relationship between fungi and plants has been the subject of extensive research, especially in the tropics, where there is a higher plant diversity and a strong positive correlation with fungi diversity. This extensive research has provided us with a wealth of knowledge about these interactions. In this study, we conducted a bibliometric analysis and systematic review, analyzing 733 research articles. A considerable growth was revealed in this field, particularly over the previous decade. Many studies during this period are concentrated in China, with a plethora of emerging researchers. More attention has been paid to genetic/molecular-based work over the last decade. In addition, researchers are promoting the use of plant–fungal interactions for sustainable agriculture, highlighting their crucial importance in mitigating crop stresses under both biotic (pests) and abiotic stresses, such as heavy metal pollutants, nutritional depletion, temperature rises, changes in water regimes, and elevated carbon dioxide concentrations. Considering future studies, further research is needed to elucidate the relationships between plants and fungi, particularly through multi-omics approaches. Network mapping and the influence of indigenous fungi on plant–fungal interactions are other, less-studied, important areas to focus on.

## 1 Introduction

Plants cannot escape exposure to microbes, from their root systems to their aboveground parts, and are always in contact with a tremendous number of microbes, including fungi, bacteria, oomycetes, protists, protozoa, and viruses (Pandey et al., 2023). A growing body of studies focuses on plant–fungal interactions, primarily examining the importance of these interactions in an agricultural environment, which ultimately affects the country’s economy (Balestrini, 2021; Dai et al., 2023). It has been estimated that the world’s fungal diversity is approximately 2.5 million species; however, only 165,000 species have been identified to date (Baldrian et al., 2022; Niskanen et al., 2023). This highlights the need for a substantial number of studies, as identifying new species is crucial to uncover further advances in the relationship between plants and fungi. According to the theoretical framework of the plant diversity hypothesis, the greater the plant diversity, the greater the microbial diversity, particularly the soil-dwelling organisms (Shen et al., 2021). It is noteworthy that studies on fungi are predominantly conducted in the tropical region; one possible reason could be the higher fungal diversity (along with the higher plant diversity). Moreover, prior studies have indicated that many cryptic species are also present in associated temperate plant species. Therefore, it is crucial to investigate the fungi present in temperate and tropical environments from the standpoint of plant–fungal interactions (Dai et al., 2023).

Fungi live with different lifestyles alongside plants, including saprophytic, pathogenic, or symbiotic. Generally, it is challenging to determine the lifestyle they may have had at the time due to the complex nature of plant–fungal interactions (Priyashantha et al., 2023). The interaction between plants and fungi is either beneficial for both, or positive for one species and negative or neutral for the other. The negative association is described in terms of pathogenicity—the disease-causing ability of fungi to their hosts. It is one of the most threatening scenarios for agriculture, responsible for up to 20% crop losses, posing significant challenges to food security in today’s world (Davies et al., 2021). However, the percentage of crop losses can also vary according to crop variety and environmental conditions, thus increasing the likelihood of severe disease incidents. Like rice blast disease, caused by *Magnaporthe oryzae*, it can generally result in harvest losses of up to 35% (Godfray et al., 2016).

In the case of extreme conditions, such as disease epidemics, production losses can exceed 60% (Różewicz et al., 2021). *Puccinia* spp. that attack wheat (*Zea mays*) can result in up to 70% crop losses, mainly due to stem rust disease (Godfray et al., 2016). In contrast, the beneficial effects of plant–fungal associations are primarily described as the promotion of plant growth and increased survivability under unfamiliar conditions (Dai et al., 2023).

Coming to the point of this study, we have employed bibliometric analysis on plant–fungal interactions related to the field of agriculture over the last 30 years. With a systematic review approach, we have comprehensively discussed the plant-symbiotic fungi interactions, emphasizing their way of creating beneficial effects for the plants. Of this, the beneficial effects of fungi in mitigating unfavorable conditions for crops, including the reduction of pests, pollutants, changes in soil nutrients, temperature rise, changes in soil water content, and carbon dioxide concentration (CO_2_) in the air, are emphasized. Then, the positive synergistic effect of different fungi on plants is discussed. Finally, challenges and required advancements in the field are briefly presented. Note that, when discussing symbiotic associations, we gave priority to the group of fungi (endophytes or mycorrhizae), which has been extensively discussed in the available literature.

## 2 Methods

A bibliometric analysis and systematic review were conducted to identify, evaluate, and synthesize studies relevant to plant–fungal interactions from a sustainable agriculture perspective. All the literature data were collected from the Scopus database^1^ from January 1, 1995, to December 31, 2024. The original research papers were only considered, excluding review papers, conference papers, book chapters, and other gray literature to avoid duplicate records. The terms searched in the Scopus search engine were— “plant–fungal association,” OR “phytopathogenic fungi,” OR “mycorrhizal fungi,” OR “plant-symbiotic fungi” AND “Sustainable agriculture.” Selected terms were filtered in the article title, abstract, and keywords. Initial search results included 1,048 papers, and in order to get the most relevant papers, further articles were filtered based on the fields such as “agriculture and biological sciences,” “environmental science,” “biochemistry,” “genetics and molecular biology,” and “immunology and microbiology” (Supplementary Figure 1). The journal published (and final stage) articles in any language were selected. The obtained papers were further checked for duplicate recordings and irrelevant records, and 315 articles were eliminated. Ultimately, 733 articles were analyzed. VOSviewer (version 1.6.20) software tool was used for bibliometric analysis and to facilitate the construction and visualization of search results. Here, co-authorship analysis, keyword analysis, and bibliographic coupling of documents were conducted.

## 3 Results

### 3.1 Publication progress, country, and language-based study projection

**Figure 1:**
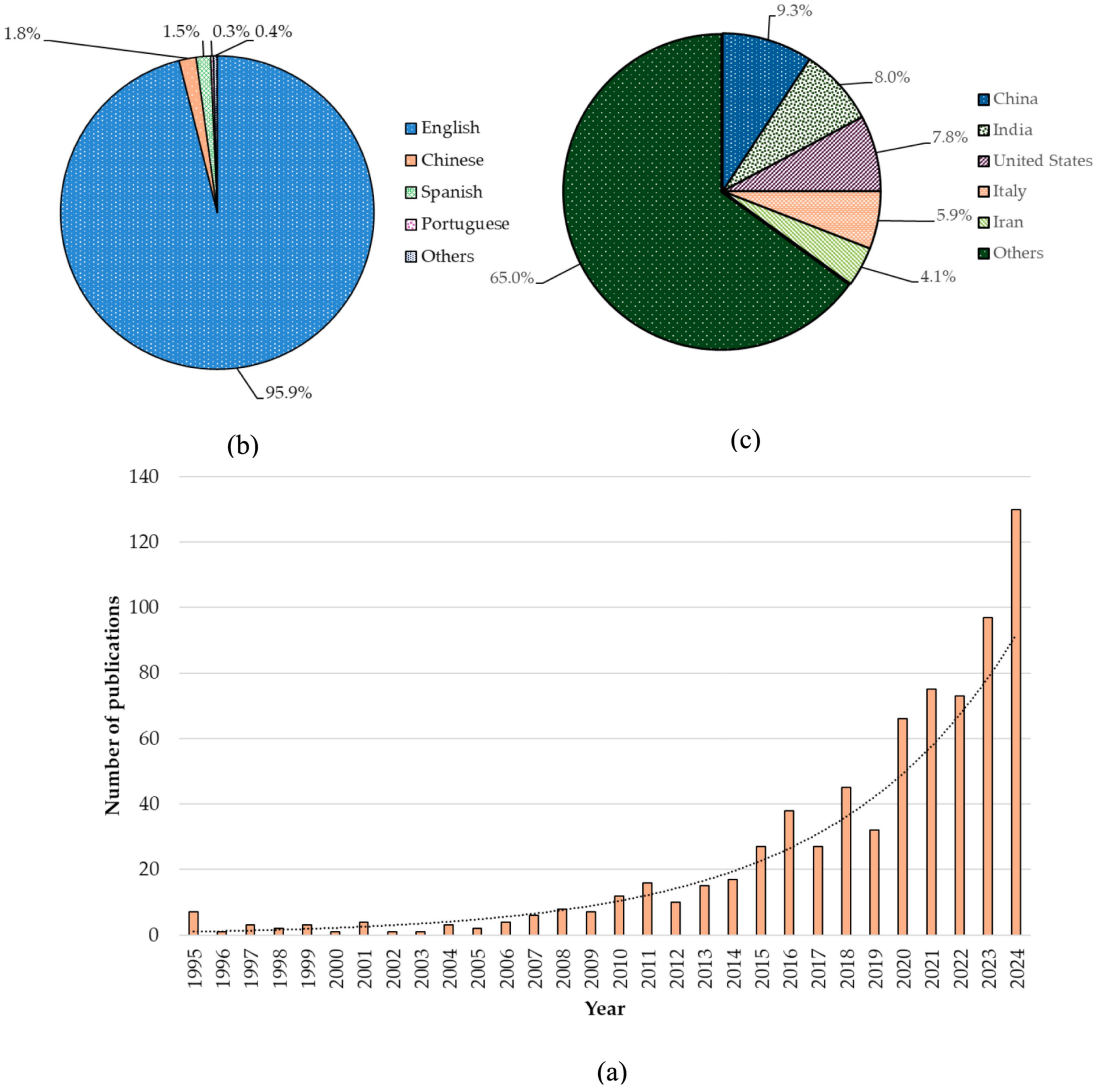
**(a)** Trends of studies during the last 30 years. **(b)** The chart shows the percentage of articles published based on language, with the following breakdown: English (95.9%), Chinese (1.8%), Spanish (1.5%), Portuguese (0.3%), and other languages (0.4%), equally shared among Polish, Persian, German, and French. **(c)** Country-wise studies percentage. Most studies were conducted in China, followed by India, the United States, Italy, and Iran, which collectively accounted for 35% of the total studies. The rest of the research was carried out by 95 other countries.

The initial data screening yielded 1,048 publications, comprising 69.9% research papers, 27.0% review articles, 1.0% editorials, 0.8% conference papers, and the remainder (1.3%), which included various types of publications such as conference papers, notes, letters, and errata. As previously mentioned, we have analyzed 733 research articles. In the first phase (1995–2004), only 26 papers were published, and during the second phase (2005–2014), 97 articles were documented in Scopus. Over the last decade, 610 articles have been published, indicating an increasing interest among researchers in this field. In detail, the most significant number of studies was recorded in 2024. Since 2010, there has been an increasing trend in the number of studies, with over 10 papers published per year. Additionally, since 2015, over 25 studies have been published annually showing the trends in this field (Figure 1c). When considering the language of published articles, approximately 96% of articles are published in English, followed by Chinese and Spanish (Figure 1b). Furthermore, the data obtained from the Scopus database indicates that publications originate from 95 countries, demonstrating a global distribution of studies. The majority of studies (68%) have been conducted in temperate or polar countries, while 32% of the work is oriented towards tropical agriculture. Among those, 9.3% of studies are coming from China, making it the leading country in research in this field. India, the United States, Italy, and Iran are among the leading countries in terms of publishing a high number of papers (Figure 1c). Moreover, our systematic review approach reveals that the lion’s share of studies focused on plant-symbiotic fungi interactions (approximately 89%), while fewer studies were conducted on plant-phytopathogenic fungi interactions (approximately 11%). However, there are no considerable trends across the continents in the studies on symbiotic versus pathogenic fungi, where the highest percentage of works are done on plant-symbiotic fungi interactions, with a more or less similar percentage of studies conducted on plant-pathogenic fungi interactions. For instance, out of 89% of studies carried out on plant-symbiotic fungi interactions, North America accounts for about 15%, while out of 11% of plant-phytopathogenic fungi interactions, it accounts for about 14%. In addition, regional *viz*, particularly a smaller number of studies conducted (less than 10) in Sub-Saharan Africa and Southeast Asia.

### 3.2 Co-authorship analysis

**Figure 2:**
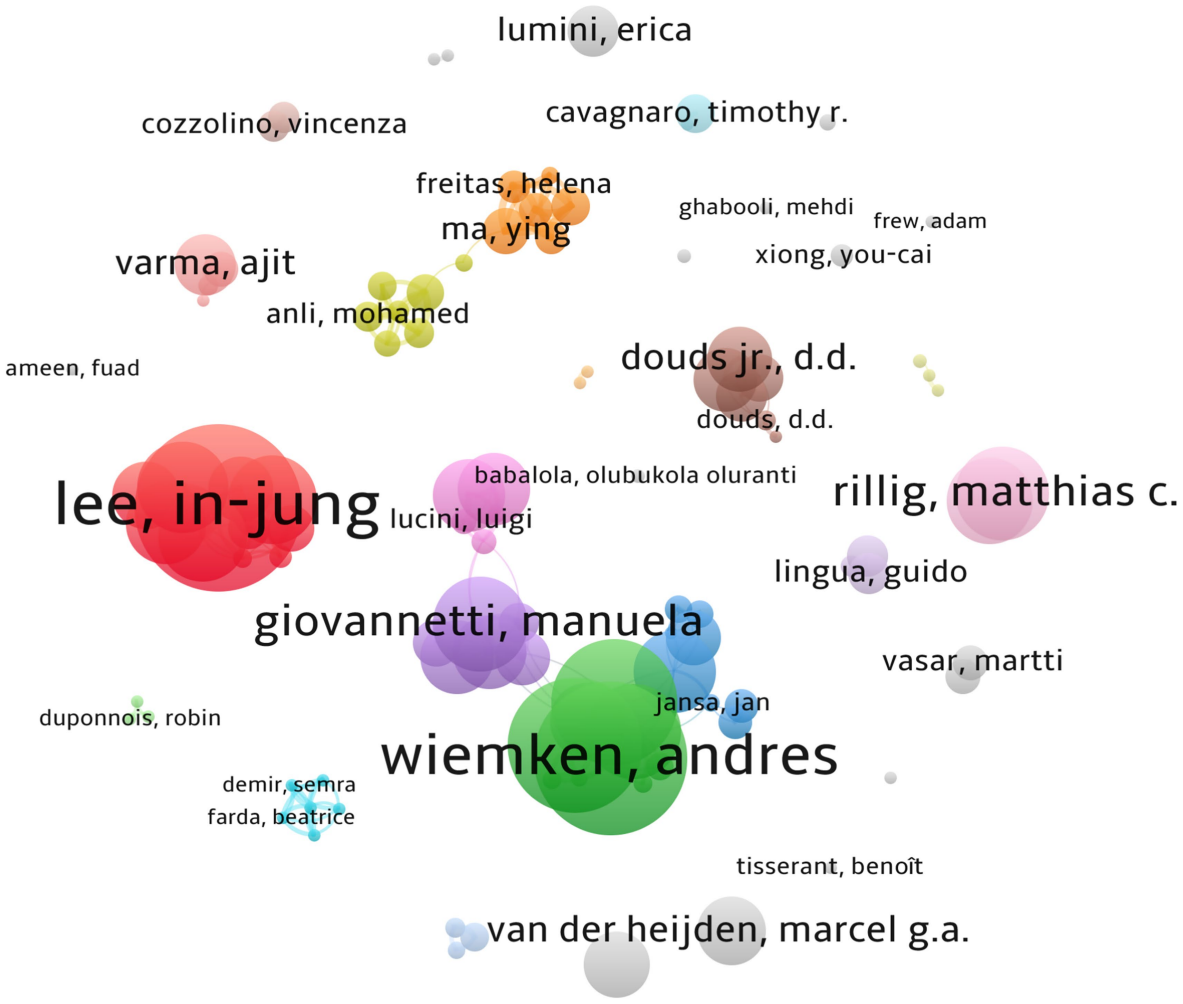
Co-authorship occurrence. A total of 104 items have met the threshold (the minimum number of authors per document is set to three), with 32 clusters, 191 links, and a total link strength (TLS) of 470. The size of the circles indicates the number of papers published by the author. The thickness of the lines represents the closeness of the collaboration between the authors.

Co-authorship analysis reveals that 3,642 scientists are actively working in this field. In Jung Lee, Muhammad Hamayun, Ying Ma, Luciano Avio, Andress Wiemken, and Helena Freitas are among the top leading scientists with the highest number of citations. Many other researchers are having collaborative works with a considerable number of citations (Figure 2).

### 3.3 Keyword analysis

**Figure 3:**
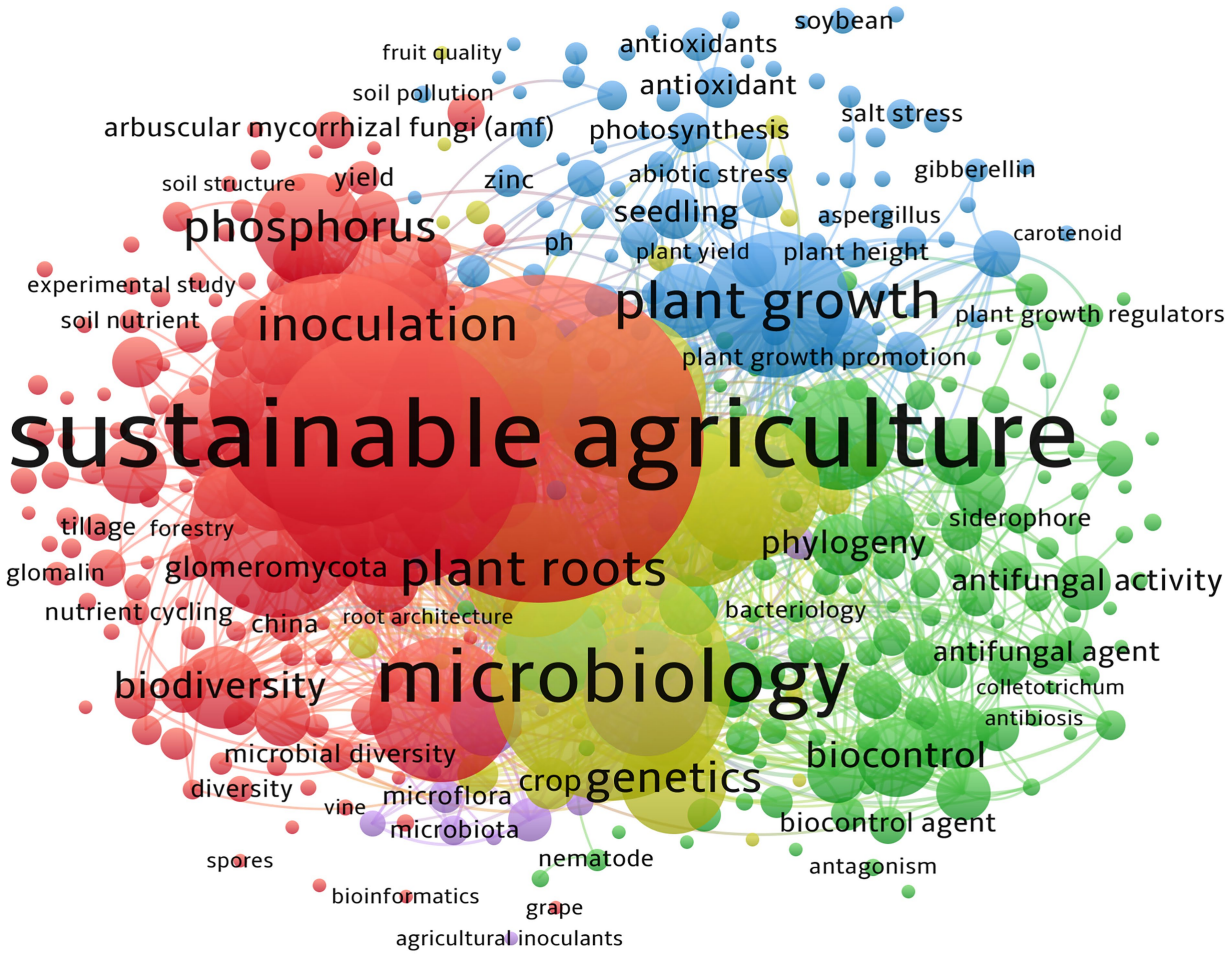
Keyword network visualization for the prominent 460 keywords. The minimum number of occurrences of keywords is set as six. The keywords are organized into six clusters, with 31,275 links, and TLS has 58,916. Note that the circle’s size corresponds to the number of keyword occurrences.

There are 5,255 keywords appeared, and according to the network co-occurrence analysis, sustainable agriculture (TLS: 2,520), mycorrhiza (TLS: 3,021) arbuscular mycorrhizal fungi (TLS: 2,065), microbiology (TLS: 3,685), alternative agriculture (TLS: 1,714), plant root (TLS: 2,405), physiology (TLS: 2,083), plant growth (TLS: 2,146), symbiosis (TLS: 1,496), soil microbiology (TLS: 2,114), metabolism (TLS: 1,983), and growth, development and aging (TLS: 1,932) are the top most keywords with occurrence of 198, 151, 148, 141, 102, 92, 86,81,81, 80,80, and 69, respectively, (Figure 3).

When the last 30 years are divided into three phases (1995–2004, 2005–2014, and 2015–2024), the dynamics of the keywords become observable. However, when considering the top five keywords, “arbuscular mycorrhiza” (also known as mycorrhiza) is the most common. Between 1995 and 2004, it was reported that 252 keywords, including sustainable agriculture, arbuscular mycorrhiza (fungi), *Zea mays*, mycorrhizae, and *Triticum aestivum*, were the most prevalent (higher TLS) keywords, with occurrences of 12, 6, 5, 4, and 4, respectively (Supplementary Figure 2A). Between 2005 and 2014, 1,292 keywords were identified, with arbuscular mycorrhiza, microbiology, mycorrhiza, alternative agriculture, and plant root being the leading keywords, occurring with frequencies of 37, 24, 24, 27, and 17, respectively (Supplementary Figure 2B). In the previous decade (2015–2024), the leading keywords were sustainable agriculture, arbuscular mycorrhiza, mycorrhiza, microbiology, and plant root, with occurrences of 168, 142, 128, 120, and 76, respectively (Supplementary Figure 2C). Moreover, there is a current trend of addressing the climatic scenario in today’s agricultural system. Here, the keyword “salinity/salt stress” (with occurrence 17) is the leading, followed by drought stress (occurrence:14). Another highlighted fact is the pollution, and heavy metal pollution is the most considered with Zinc (Zn) pollutants (occurrence: 14).

### 3.4 Bibliographic coupling of documents

**Figure 4:**
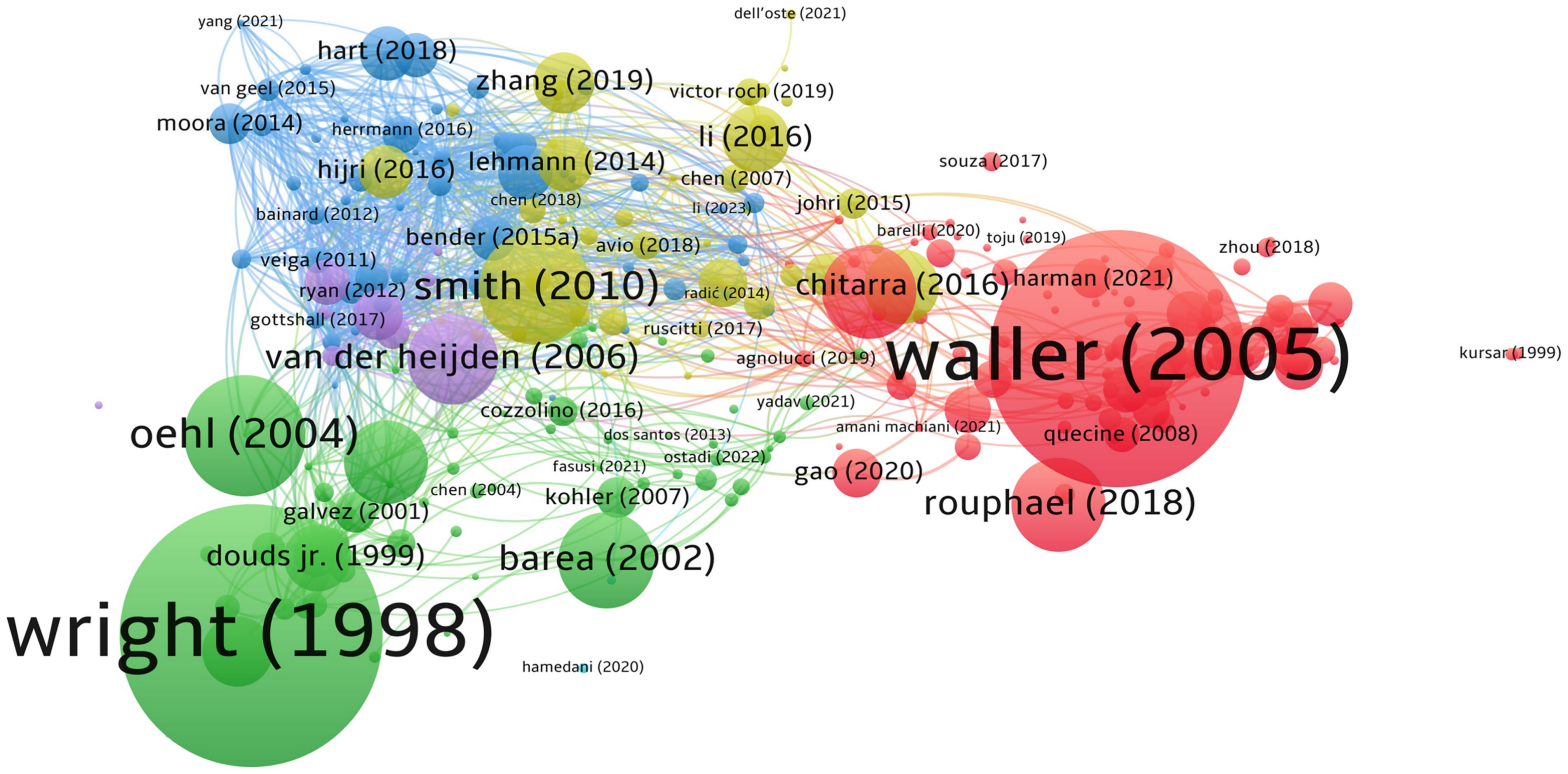
Bibliographic coupling of documents. Out of 733 documents, 279 met the threshold limit of 20 citations, with six distinct clusters, 8,964 links, and 16,196 TLS. This figure represents that the higher the weight citation, the larger the circle. The lines show the relationship links between the articles. Note that, first author of each document only shows in here.

The bibliographic coupling shows the usefulness of measuring thematic similarities and relations of publications. The documents by Wright and Upadhyaya (1998) and Waller et al. (2005) are strongly coupled (Figure 4). The cohorts of their studies concentrated on arbuscular mycorrhizal and endophytic fungi, respectively. Other highly bibliographic couplings, as shown by Oehl et al. (2004), discussed the influence of terms, including conventional and organic farming, on the diversity of arbuscular mycorrhizal fungi. Nevertheless, considering the bibliometric analysis outputs, Pellegrino (2014), Bedini (2013), Elliott (2021), Kohil (2016), and Bainard (2012) represent the top five based on TLS of 501, 437, 418, 402, and 399, respectively.

## 4 Discussion

**Figure 5:**
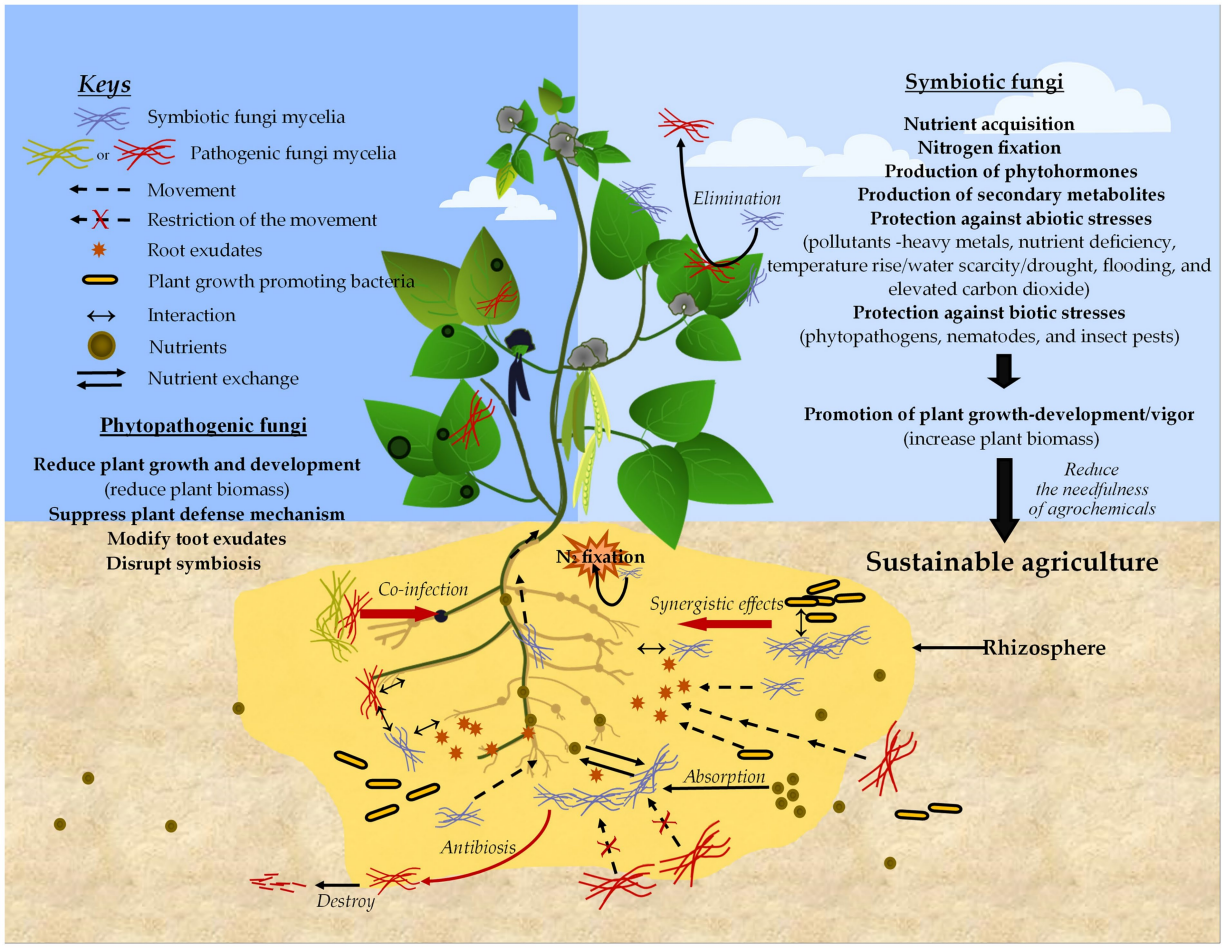
Simplified schematic diagram of plant–fungal interactions illustrating the positive and negative impacts on plants, as well as their contributions to sustainable agriculture.

As previously highlighted, the interaction between plants and fungi is one of the most prominent factors in a healthy or sustainable agricultural system. Phytopathogenic fungi are nightmares to the agriculture sector, while symbiotic fungi are a pleasant dream (Figure 5). Through data analysis, we have also found that approximately 5.4% of studies directly focused on plant–fungal interactions with climate-resilient or low-input agricultural systems, while the majority of other studies indirectly highlighted this. Although highly developed techniques exist to eliminate phytopathogenic fungal diseases, the current world is still unable to eradicate them. Therefore, it is still vital to discuss those fungal interactions with the plants. On the other hand, plant-fungal interactions in the perception of some of the world’s major crops (e.g., cassava, cotton, potato, sorghum, soybean, sugarcane) need to be more thoroughly studied.

Studying the plant–fungal interactions are also today more focused on molecular biology. The molecular trends in plant–fungal interactions encompass the intricate signaling pathways and gene expression patterns that govern both beneficial and detrimental relationships between plants and fungi. Although this is not our primary focus in this paper, it is essential to briefly highlight this aspect. In the current trend, the involvement of pathogenic fungi in regulating pathogenicity through surface signal recognition proteins, Mitogen-Activated Protein Kinase (MAPK) signaling pathways, transcription factors, and pathogenic factors during plant infection is being explored by researchers (Li et al., 2022). Pathogenesis-related (PR) proteins, encoded by *PR* genes, accumulate in response to pathogen attack and play a direct role in plant disease resistance. The development of a hypersensitive response (HR) is another protective reaction provided by plants, which can induce the systemic acquired resistance (SAR) mechanism (Balint-Kurti, 2019), and is still receiving considerable attention among researchers (Ali et al., 2018). In addition, the plant’s resistance mechanism is also induced by the symbiotic fungi in a manner that is beneficial in fighting against pathogens (Constantin et al., 2019). A plethora of recent studies also focused on further understanding of plant innate immunity immune responses in plant-fungal interaction, namely: pattern-triggered immunity (PTI) and effector-triggered immunity (Naveed et al., 2020; Percio and Botella, 2025).

It should also be noted that the interaction between plants and fungi can differ in natural and agricultural ecosystems. In natural ecosystems, such as forests, fungal diversity and richness can be significantly higher than those found in agricultural lands (Öpik et al., 2006). Obviously, this is due to the introduction of various agrochemicals (and other farming practices), leading to shape up of the fungal population, thus influencing their association with plants. In natural ecosystems, symbiotic associations are often more prominent, and there is generally a dynamic balance between symbiotic and pathogenic fungi. In contrast, in an agricultural system, changes in farming operations can significantly alter the fungal population, ultimately affecting plant-fungal interactions (Zeilinger et al., 2016; Priyashantha et al., 2025).

### 4.1 Phytopathogenic fungi

Based on the mode of nutrition, pathogenic fungi have been classified into three main categories namely, biotrophs (obtain nutrients and energy from living cells), necrotrophs (derive energy from dead or dying cells), and hemibiotrophic (derive nutrients initially from living cells and later transferred into necrotrophic lifestyle and take up nutrients from killing the cells) (Rajarammohan, 2021). All these pathogenic groups may develop diseases following contact with plants; however, this is not always the case. To cause a disease, it requires the presence of specific conditions, as explained in the famous classical disease triangle, which posits that a disease is caused by the combination of three main factors: a susceptible host, a virulent pathogen, and favorable environmental conditions. Elimination of one of those three components will not cause disease to the plants (Gaumann, 1950; Stevens, 1960). Nevertheless, this has been further explained genetically and refined when describing the host-pathogen interaction, as the genotypes of the pathogen and the host, along with environmental factors, combine to determine the outcome of this association (Casadevall and Pirofski, 1999, 2000). Plant survival and pathogen detection also influenced the pathogen-host–environment. The spatial and temporal distribution of pathogens concerning the host depends on the biology of the pathogens, including aspects of pathogen dispersal and overwintering capacity (Benítez et al., 2013). According to the bibliometric analysis, most studies have focused on single-host-single-disease interactions, with less attention given to co-infections. However, by understanding this, today, co-infection is becoming an interesting point for the researchers. Among these, works conducted by Lerch-Olson and Robertson (2020), Fang et al. (2021), and Zhang et al. (2022) can be cited. Host-pathogen, pathogen–pathogen, and host-multiple-pathogen complexes are the three main interactions that cause damage in co-infected plants. Host–pathogen interaction is the most extensively studied, while the other two interactions are relatively obscure. These interactions can yield various outcomes, including antagonism, synergism, coexistence, mutualism, or cooperation (Abdullah et al., 2017). The development of disease due to co-infection remains unresolved, and the crucial factors that determine the outcome of co-infections also remain unclear (Barrett et al., 2021). Nevertheless, in general, co-infection with fungi can have a substantial adverse effect on the host plant, making it more susceptible to diseases. Moreover, the co-occurrence of several fungal species could lead to disease outbreaks (Zhou et al., 2023).

### 4.2 Symbiotic fungi

Mycorrhizae and endophytes are two main groups of beneficial fungi that have been mostly studied. Endophytic fungi are facultative plant symbionts or biotrophs, and unlike mycorrhizal fungi, their development is not synchronized with that of their respective hosts (Domka et al., 2019). Endophytic fungi primarily colonize the intercellular or intracellular spaces of plants. In addition to the presence of endophytic fungi in aerial parts such as fruits, leaves, peduncles, seeds, and stems, they are primarily associated with the underground parts of the plants (Asad et al., 2023). Among several other mycorrhizal types, arbuscular mycorrhizal fungi (AMF) are the prominently studied group, emphasizing their tremendous benefits towards agriculture. Unlike plant-pathogenic fungi, which cause host destabilization, beneficial fungi provide stability to the host. The interaction of endophytic fungi can alter the metabolic activities of the plants (Usman et al., 2024), supply more nutrients, and protect them from harmful pests, thus plants show improved growth and development, and strengthening the plant against various biotic factors such as high temperature, drought, salinity, acidity, and waterlogged (Zeilinger et al., 2016; Akhtar et al., 2022). Under stressed conditions, the interaction of these symbiotic fungi is crucial, as they produce various stress-responsive molecules. Among these, symbiotic fungi stimulate the production of plant hormones, including gibberellins (GB), cytokinins (CIS), salicylic acid (SA), and indole-3-acetic acid (IAA) (Singh et al., 2023). Furthermore, symbiotic fungi can also produce such hormones and improve support to the plants in mitigating stress conditions (Ikram et al., 2018). Additionally, symbiotic fungi usually enhance the plant synthesis of defense enzymes, antioxidant activity, and expression of SA- and Jasmonic acid (JA)-responsive genes. Furthermore, they minimize reactive oxygen species (ROS) and reactive nitrogen species (RNS), and enhance callose deposition, which is beneficial against pathogenic fungi (Morelli et al., 2020). However, researchers have also found that endophytic fungi can exhibit a distinct pathway of making host plants resistant to phytopathogens, such as endophyte-mediated resistance (EMR). Here, according to the findings of Constantin et al. (2019), the EMR is independent of JA/ET and SA-dependent signaling pathways and distinct from the classical induced systemic disease resistance. Their study implies that antibiosis, competition for niches or nutrients, or a plant response independent from SA, ET, and JA are involved in EMR. Nevertheless, Matušinsky et al. (2022) suggested that the cooperative effect of the induced systemic disease resistance mechanism and EPR exists. They have highlighted the key genes involved in creating disease resistance in studied organisms—*B2H2*, *Chitinase*, *LOX*, *PR1*, and *PR1*.*1*. Recently, a literature review conducted by de Lamo and Takken (2020) provided a comprehensive explanation of EMR and also reported a molecular-level understanding of EMR.

### 4.3 Beneficial aspects of plant-symbiotic fungi towards sustainable agriculture

When it comes to agriculture, plant–fungal interactions are highly influenced by farming practices. The effect may be both direct and indirect, causing either the death of fungi or improved colonization (Begum et al., 2019). Nevertheless, in general speaking, farming practices decrease the natural fungi populations, also dropping the species diversity. However, when compared with different farming systems and conventional and organic practices, the growth of microbial biomass and its diversity is influenced by conventional practices (Manoharan et al., 2017). The farming practices led to changes in the soil’s nutritional regime; moreover, modern farmlands are mostly polluted by various hazardous compounds. In addition to the farming practices, climatic changes contribute to significant changes in plant–fungal associations, due to alterations in temperature, light, water availability, and CO_2_ concentration (Singh et al., 2023). One of the most intriguing outcomes of the alterations of the factors mentioned above is the lifestyle shift of the fungi. More precisely, these factors may lead to a shift from a pathogenic to a mutualistic lifestyle and vice versa (Priyashantha et al., 2023). Theoretically, the emergence of new pathogenic strains is associated with increased virulence in overcoming host-plant resistance (Singh et al., 2023). Nevertheless, plants may also be exposed to novel effectors despite having extensive defense mechanisms in place, due to the rapid evolution of pathogens, which could also be attributed to changes in environmental factors (Möller and Stukenbrock, 2017). In the case of symbiotic fungi, their importance in agriculture is highly valuable. Moreover, the use of native symbiotic fungi inoculum in agriculture may be more beneficial than the application of exotic species; however, this has not been the primary focus of many studies (Klironomos, 2003). Nevertheless, our aim here is to discuss the significance of plant–symbiotic fungal interactions in addressing both biotic and abiotic stresses.

#### 4.3.1 Pests

Along with diverse bioactive metabolites produced by symbiotic fungi, this creates the possibility of controlling a wide range of pests (Wang et al., 2023), including plant-parasitic nematodes and herbivorous insects (Akram et al., 2023). When mycorrhizal and endophytic fungi are used as biocontrol agents, they yield inconsistent results, including both negative and positive outcomes among studies. This could be due to the condition of the tested environment and differences in the techniques and methodologies followed; however, it is crystal clear that fungi can control phytopathogens and improve plant health (Table 1). On the other hand, most of the works conducted by researchers are limited to the control environment/laboratory scale, and the need for field evaluation needs to be highlighted (Suebrasri et al., 2020; Azuddin et al., 2021; Mota et al., 2021). In a study, Yanan et al. (2015) recognized the enhanced resistance of strawberry plants when inoculated with *Glomus mosseae* against Fusarium wilt caused by *Funneliformis mosseae*. The authors found that an increase in cell wall lignin and HRGP (hydroxyproline-rich glycoprotein) proteins, along with changes in the activities of catalase, peroxidase, and superoxide dismutase enzymes, leads to such resistance. Wang et al. (2022a) emphasized the importance of *Rhizophagus irregularis* against *F. oxysporum*, as they trigger the plant defense mechanism, induce expression of the JA synthesis genes, such as allene oxide cyclase gene (*AOC*) and lipoxygenase D gene (*LOXD*), and increase activities of polyphenol oxidase (*PPO*) and phenylalanine ammonia lyase (*PAL*). Khruengsai et al. (2021) showed that *Daldinia eschscholtzii* MFLUCC 19–0493 isolated from *Barleria prionitis* inhibits the anthracnose disease fungi *Colletotrichum acutatum,* through the production of various other volatile compounds—3,5-dimethyl-4-heptanone, benzaldehyde dimethyl acetal, elemicin, ethyl sorbate, methyl geranate, and trans-sabinene hydrate. In contrast to previous works, Odunayo Joseph and Victor Olumide (2022) aimed to identify the additional benefits provided by the fungi beyond pest resistance. Odunayo Joseph and Victor Olumide (2022) showed the importance of *Glomus clarum* in controlling the tomato early blight disease caused by *Alternaria solani*. Their results showed that once inoculated with *G. clarum*, the plants exhibited enhanced resistance to the pathogenic fungus. Moreover, with the enhanced nutrient supply, the crop showed increased production of flowers, fruit weight per plant, leaf condition, and stem growth.

**Table 1:** Recent study findings show the pest-controlling ability of plant symbiotic fungi.

Fungi	Host plant	Controlled pests (Fungal pathogens = FP, Bacterial pathogens = BP, Nematodes = Nem)	Name of the pathogen/pest	Main findings	References
Mycorrhizal fungi (AMF)					
*Acaulospora longula*, *Claroideoglomus claroideum, Glomus intraradices,* and other indigenous mycorrhizal fungi.	Apple (*Malus domestica*)	Nem	*Pratylenchus penetrans*	The dual-species application of AMF biocontrols nematodes significantly (max. 68%) and also provides additional nutrients to the host plant. The greatest biocontrol is achieved (97%) when using a mixture of 13 other indigenous mycorrhizal fungi.	Ceustermans et al. (2018)
*Ambispora* sp., *Funneliformis* sp., and *Glomus* sp.	Grapevine (Vitis sp.)	PF	*Dactylonectria macrodidyma, Ilyonectria europaea,* and *Ilyonectria liriodendri*	The AMF significantly minimize disease incidents (40 to 50%). In addition, fungi also increase plant growth.	Moukarzel et al. (2022)
*Claroideoglomus etunicatum*	Lemongrass (*Cymbopogon citratus*)	Nem	*Pratylenchus brachyurus*	The fungus promotes an increase in shoot weight, preserves the essential oil composition, and enhances polyphenol oxidase activity in nematode-infected plants. However, the AMF does not result in a decrease in nematode population density.	Rodrigues E Silva et al. (2021)
*Funneliformis mosseae, Glomus monosporum,* and *Rhizoglomus clarum* mixture.	Garlic (*Allium sativum*)	FP	*Sclerotium cepivorum*	The mixture of AMF controls pathogen infection by triggering the plant’s resistance mechanism, improving plant nutrition, growth, and stimulating the accumulation of photosynthetic pigments. It also enhanced the accumulation of antimicrobial substances, such as phenolic compounds and defense-related enzymes, and activated certain defense-related genes.	Rashad et al. (2020a)
*Claroideoglomus* sp., *Diversispora* sp., *Funneliformis* sp., *Glomus* sp., and *Rhizophagus* sp.	Watermelon (*Citrullus lanatus*)	FP	*Rhizoctonia solani*	The AMF reduces the hydrogen peroxide content and the accumulation in host plant roots. The fungi also significantly increased the root activity and relative electrolyte leakage under pathogenic stress. Furthermore, the AMF increases the biomass of the plant, while a higher colonization rate of AMF leads to greater promotion of plant growth. Also, AMF composition consisting of distant species may give better results than a single application or closely related species.	Wu et al. (2021a)
*Glomus versiforme* and *Glomus mosseae*	*Wedelia trilobata*	FP	*Rhizoctonia solani*	The fungi promote the growth and disease resistance of *W. trilobata* under a nutrient-poor environment.	Chen et al. (2021)
*Rhizophagus clarus*	Lemongrass (*Cymbopogon citratus*)	Nem	*Pratylenchus brachyurus*	The fungus preserves the essential oil composition and enhances polyphenol, peroxidase, and oxidase activity in nematode infected plants. However, the fungi do not have a significant effect on reducing the population density of the nematode.	Rodrigues E Silva et al. (2021)
*Rhizophagus irregularis*	Sunflower (*Helianthus annuus*)	FP	*Rhizoctonia solani*	The AMF improved growth and reduced disease severity. This may also occur due to the induced plant resistance mechanism, which involves cell wall lignification, thereby enhancing the accumulation of chlorogenic acid, flavonoids, and anthocyanins.	Rashad et al. (2020b)
Not specified	Rice (*Oryza sativa*)	BP	*Xanthomonas oryzaepvoryzae*	The fungus significantly reduces the disease, resulting in the lowest disease intensity (16.51%). The fungus also significantly improves plant height and tiller density.	Marlina et al. (2020)
Endophytic fungi					
*Chaetomium globosum, Echria macrotheca,* and *Stagonospora trichophoricola*	Chili pepper (*Capsicum annuum*) and tomato (*Solanum lycopersicum*)	Nem	*Meloidogyne enterolobii, M. incognita,* and *Nacobbus aberrans*	The fungi considerably controlled the nematodes. *Chaetomium globosum* parasitized eggs of *M. enterolobii*, while *E. macrotheca* and *S. trichophoricola* parasitized the eggs of *N. aberrans*. Further, *E. macrotheca* affects the eggs of *M. incognita* and the juvenile (J2) of *N. aberrans*. All the endophytic fungi promote the growth of chili peppers, while *S. trichophoricola* also enhances the growth of the tomato plant.	Silva-Valenzuela et al. (2023)
*Dichotomopilus* sp., *Epicoccum* sp., *Microdochium* sp., and *Schizothecium* sp.	‘Cinnamon Girl’ pumpkin (*Cucurbita pepo*)	PF	*Verticillium dahliae*	The endophytic fungi do not significantly impact foliar disease severity or fruit yield in the field experiment. However, recovery of colony-forming units of pathogens (from pumpkin stem sap) is significantly lower for plants inoculated with either *Dichotomopilus* sp. or *Schizothecium* sp. When tested in the greenhouse, endophytic fungi significantly increased plant vigor, fresh weight, root fresh weight, and root dry weight.	Tymon et al. (2020)
*Fusarium solani*	Cotton plant (*Gossypium* sp.)	PF	*Verticillium dahliae*	The dual culture test showed that the endophytic fungus inhibited the mycelial growth of *V. dahliae* by an average of 75%. *Fusarium solani* also significantly reduces the sporulation (nearly 80%) and wilt disease development in the greenhouse experiment. Even in the field, the disease index is significantly reduced with the inoculation of endophytic fungi (from 30.1 to 56.3%). Furthermore, the *PR* gene, a key gene in the lignin metabolism pathway, is transiently upregulated in *F. solani.*	Wei et al. (2019)
*Fusarium solani,* and *Trichoderma asperellum*	Chilli pepper (*Capsicum annuum*)	BP	*Ralstonia solanacearum*	The fungi control the bacterial wilt disease with inhibition rate of 12.5 to 50%. Further, the combined application of both fungi does not always suppress the disease development. The fungi, whether applied individually or in combination, did not show a significant effect on plant growth. Especially, *F*. *solani* showed an increased activity of plant compounds such as total phenol, peroxidase, polyphenol oxidase, *β*-glucanase, and phenylalanine aminaliase.	Irawati et al. (2020)
*Trichoderma atroviride* and *Xylaria adscendens*	Tahiti lime (*Citrus citrus* × *latifolia*)	FP	*Colletotrichum acutatum*	In vitro tests resulted in a significant reduction in disease caused by phytopathogens. The disease severity test showed less than 5% lesions in flowers.	Muñoz-Guerrero et al. (2021)

In their *in vitro* assay, Suebrasri et al. (2020) showed that several endophytes, *Diaporthe phaseolorum* BUP3/1 and *Macrophomina phaseolina* BUP2/3, isolated from Jerusalem artichoke (*Helianthus tuberosus*) and *D. eschscholtzii* 2NTYL11 and *Trichoderma erinaceum* ST-KKU2, isolated from Stemona root (*Stemona tuberosa*) and ginger (*Zingiber officinale*), control the stem rot fungi (mycelial growth) *Sclerotium rolfsii* impressively at 76.00, 41.20, 66.67, and 63.63%, respectively. Furthermore, the pot experiment showed that *T. erinaceum* ST-KKU2 and *D. eschscholtzii* 2NTYL11 control the disease at a higher level (58.14%) than the others. Furthermore, they have observed predatory mechanisms, and the production of polyketide groups (6-n-pentyl-2H-pyran-2-one/6PAP and 2,3-dihydro-5-hydroxy-2-methyl-4H-1-benzopyran-4-one/DHMB) is a primary factor in controlling the pathogens. Many other studies have also shown the effects of various endophytic fungi in significantly controlling major devastating phytopathogens in crops, e.g., *Induratia coffeana* inhibits *Colletotrichum lindemuthianum* at 99.64 ± 0.57 (Mota et al., 2021); *T. harzianum* inhibits *Colletotrichum scovellei* at 85.80 ± 5.47 (Azuddin et al., 2021); and *Annulohypoxylon* sp. inhibits *Penicillium digitatum* at 72.96 ± 0.58 (Du et al., 2022). The symbiotic fungi also produce various antibacterial compounds, such as acetic acid, acetol, and hexanoic acid, among others (Rashid, 2021). Similarly, a number of compounds produced by symbiotic fungi also control the plant parasitic nematodes, for example, 3-methoxyepicoccone, 4,5,6-trihydroxy-7-methylphthalide, chaetoglobosin A, chaetoglobosin B, and flavoring isolated from *Chaetomium globosum* YSC5 found to be working against *Meloidogyne javanica* (Khan et al., 2019). The application of endophytic fungi also exhibits good nematocidal effects due to these compounds. In a study by Yao et al. (2023), it was found that *Acremonium sclerotigenum* effectively controls *M. incognita*, with a mortality rate of up to 95.5% in the juvenile (J2 stage) and significant inhibition of egg hatching of up to 43%. Schouteden et al. (2015) emphasized the importance of AMF in controlling nematodes. Adding value to this, Bell et al. (2023) highlighted the effectiveness of various AMFs in controlling potato cyst nematode *Globodera pallida*. In turn, the importance of symbiotic fungi against plant viruses is still less of a concern. However, available studies have shown their significance (Fakhro et al., 2010; Jaber and Salem, 2014; Kiarie et al., 2020).

It is also important to note that the majority of studies have focused on the application of endophytic fungi to control insect pests, rather than mycorrhizal fungi, despite showing their undeniable importance (Vannette and Hunter, 2009; Jiang et al., 2021). Control of insect pests using fungi has a long history, dating back to the 1980s. *Beauveria bassiana* is the earliest identified and most recognized entomopathogenic endophyte in controlling a wide range of pests (Islam et al., 2023). Today, in addition to *B. bassiana*, several other entomopathogenic fungi, including *Metarhizium, Isaria,* and *Lecanicillium*, have been used to control insect pests. However, such utilization is still in its early stages of development and requires considerable further study, particularly in identifying new infectious fungal species and strains (Bamisile et al., 2023). Suebrasri et al. (2020) examined the effect of *B. bassiana* and *Purpureocillium lilacinum* against the cotton aphid, *Aphis gossypii*. They have recognized that fungi have an impact on the host’s reproduction. They have found the significant control of aphids by *B. bassiana*, while *P. lilacinum* shows considerable but not significant control. Agbessenou et al. (2020) studied the controlling ability of Lepidopteran *Tuta absoluta* using tomato (*Solanum lycopersicum*) and nightshade (*Solanum scabrum*) *Trichoderma asperellum* M2RT4, *B. bassiana* ICIPE 706, and *Hypocrea lixii* F3ST1. They have found the effectiveness of those fungi in reducing the number of eggs laid, mines developed, pupae formed, and adults emerged. In contrast to the above two studies, Pappas et al. (2018) recognized the indirect control of pests by endophytic fungi in their study on *Fusarium solani* strain K against two-spotted spider mites (*Tetranychus urticae*) in tomato. They have studied the expression of genes *GGPS1, JIP-21*, *LOXD*, *PPO-D*, *PPO-F*, *PR-1A*, *PR-P6*, and *WIPI-II* in the pest after fungi colonization, which is important for host defense mechanisms against pests. However, according to their findings, the fungi significantly upregulated the expression of WIPI-II and PPO-D. At the same time, no effects were observed on other mite-defense-related genes, such as *GGPS1*, *JIP-21*, *LOXD*, *PPO-F*, *PR-1A*, and *PR-P6*. The indirect effect was also recognized through the alteration of the plant’s volatile compound emissions, such as decanal, 5-heptene-2-one-6-methyl, and geranyl acetone.

#### 4.3.2 Pollutants

Heavy metal pollutants such as arsenic (As), cadmium (Cd), cobalt (Co), copper (Cu), lead (Pb), magnesium (Mg), manganous (Mn), nickel (Ni), strontium (Sr), and zinc (Zn) are known to be a major threat to agriculture today. The excessive release of heavy metals into the environment leads to biological toxicity and direct environmental pollution (Borymski et al., 2018; Asiminicesei et al., 2024). Furthermore, those metals that are injurious to plants also alter metabolite activities and reduce total productivity (Jańczak-Pieniążek et al., 2022). Moreover, it has been understood that heavy metals impede seed germination, shorten roots and shoots, and lower respiration and photosynthesis rates (Majhi and Sikdar, 2023). A major problem occurs when crops absorb heavy metals, and human consumption of these contaminated crops leads to life-threatening diseases (Boluspayeva et al., 2022). In early studies, microbes and plants have been used separately to eliminate those heavy metal pollutants from the soil prominently; nevertheless, understanding the importance of plant–fungal interactions, combined application of both plants and fungi became a catchier topic nowadays (Flemming et al., 1990; Joshi-Tope and Francis, 1995; Boorboori and Zhang, 2022). The plant-based approach to removing heavy metals, known as phytoremediation, involves the application of selected species that are resistant to heavy metals and accumulate high levels of metal (loid)s in their plant bodies, primarily in the roots and shoots. The plants are selected based on the two assignments: bioconcentration factor (BCF) and translocation factor (TF). The shoot-to-root ratio of heavy metal and the root-to-soil ratio of heavy metal (Sharma et al., 2023).

In general, vascular plants typically have a marginally higher tolerance to heavy metals (Fasani et al., 2022). Plants can alter the number of polysaccharides in their cell walls to withstand the stress caused by heavy metals. The main proteins in cell walls that are involved in cell wall dynamics and plant responses to stressors are called peroxidases. As plant roots grow, they release secretions into the soil, including low-molecular-weight organic acids, which break down soil minerals and release metals (Boorboori and Zhang, 2022). Mycoremediation, the removal of heavy metal stress by fungi, could be a more effective approach because fungi can move across and penetrate toxic or unfavorable zones to reach substrates and nutrients (Antón-Herrero et al., 2023). The underlying mechanism for mitigating the heavy metal threats is not yet well understood (Fan et al., 2022). Nevertheless, according to the findings, the homeostatic systems of fungi regulate the import, export, storage, and transport of heavy metals. It is also known that oxalate crystals produced by mycorrhiza fungi immobilise and detoxify heavy metals. The filamentous hyphae of these fungi enter the deeper soil aggregates and chelate or adsorb heavy metals (Mishra et al., 2017). In more detail, for example, ATP-binding cassette (ABC) transporters regulate several heavy metals, including Cd (Víglaš and Olejníková, 2021); CtrA2, CtrC, and CtrB transporters coordinately work on Cu (Raffa et al., 2019); and SMF1 and SMF2 function on Mn (Mori et al., 2018). Cytoplasmic compounds in fungi transform hazardous metals into less or non-toxic forms, which can then be segregated within the vacuole. Metallothioneins (MTs), a major class of intracellular peptide chelators of metal ions, play a crucial role in cellular tolerance to and detoxification of heavy metals (Gajewska et al., 2022). Moreover, it has been recognized that fungi’s genetic mechanisms of adaptation, or modifying their expression patterns, result in different profiles of gene expression (Traxler et al., 2022). The *MT* genes of fungi can be induced even by a single heavy metal, triggering the detoxification mechanism (Gajewska et al., 2022).

Lorenzo-Gutiérrez et al. (2019) demonstrated the role of *mt1* in conferring tolerance to *F. oxysporum* against Cd, Cu, and Zn. Gautam et al. (2023) recently showed the metal-binding and heavy metal tolerance mechanisms of metallothionein *OsMT-I-Id*. Furthermore, their evidence shows the ability of fungi to induce *OsMT-I-Id* in rice against As, Cd, and Cu. It has also been identified that other genes, particularly *ChrA*, *ChrB*, and *ChrR*, function in response to a specific heavy metal. Those genes facilitate the fungi to tolerate Cr pollutants (Chi et al., 2021; He et al., 2021). In a recent study, Chi et al. (2021) analyzed the genome of P1 in *Penicillium janthinellum* and annotated 23 genes related to heavy metals.

Substantial investigation has been conducted to examine the significance of fungi in tolerating/degrading heavy metals. Ahmad et al. (2006) found that *Aspergillus niger* (also *Penicillium* sp.) tolerates heavy metals like Ni, Cr, and Cd. They have further recognized that fungi tolerance is not just under single-metal conditions but also under multi-metal conditions. Acosta-Rodríguez et al. (2018) later found the tolerability of *A. niger* against 2,000 ppm of Cr, Zn, Pb, and Hg, 1,200 and 1,000 ppm of Cu, As (III) and (VI), 600 ppm of Co, and 400 ppm of Cd.

Further, their study evident that, for instance, the ability of the fungus to eliminate Zn and Cr (VI) by 100%, followed by Hg (83.2%), Co (71.4%), As (V) (69%), As (III) (66%), Pb (59%), Cd (57%), and Cu (37%). In addition, Acosta-Rodríguez et al. (2018) gave an in-depth understanding of the mycoremediation potential of fungi. They highlighted that *A. niger* is not always superior in removing heavy metals, as it may remove specific metals (and certain concentrations) that could also depend on temperature and pH of the substrate. In a separate study, Mohamadhasani and Rahimi (2022) found that *Pleurotus* species grew more under low concentrations of the tested heavy metals (Co and Cu), indicating potential for heavy metal removal. It is noteworthy that when a fungus-like AMF establishes a direct relationship with plants, it increases the immobilization, conversion, detoxification, and extraction of heavy metals (Boorboori and Zhang, 2022). It is also recorded that a wide range of AMF can withstand highly polluted soils, thus, offering future possibilities for finding novel AMF species with greater mycoremediation ability (Suárez et al., 2023).

In a study, Iram et al. (2019) used several *Aspergillus* spp. (*A. niger*, *A. terreus*, and *A. flavus*) and *Penicillium* (*P. chrysogenum*) to remediate the Cr, Cu, Pb, and Cd associated with wheat and sunflower (*Helianthus annuus*). Plant–fungal interaction significantly helps remove the tested contaminants, enhanced by Aspergillus sp. (Cr < Cu < Pb < Cd). Furthermore, the researchers found that the interaction of *P. chrysogenum* leads to the storage of a higher amount of Pb in the plant shoot compared to other metals. Fan et al. (2022) found that *A. niger* (TL-F2) and *A. flavus* (TL-F3) facilitated the accumulation/subcellular distribution of Cd by annual ryegrass. Further, the researchers observed that ryegrass roots (17.8–37.1 μg pot^−1^) were significantly more capable of absorbing Cd than the shoots (1.66–5.45 μg pot^−1^). Overall, it was observed that the accumulation of Cd in different subcellular fractions increased with Cd concentration from 1.96 to 10.2 mg kg^−1^. Nevertheless, compared with non-fungus ryegrass, cell wall and soluble Cd fractions in fungus-inoculated roots increased by 13.5–44% and decreased by 21.5–26.4%. Additionally, the authors recognized that fungal interaction significantly increases the plant’s biomass. For example, the biomass increases of fungus-inoculated roots range from 14 to 43%. Regarding the increasing Cd solution, in non-fungus-inoculated plants, the dry biomass is decreased by 10–40%. Chen et al. (2007) demonstrated the role of *F. mosseae*, in removing the effect of Phosphorus (P) and As associated with alfalfa. They inoculated the fungus and observed the increase in dry weight of both shoots and roots of the plants. Furthermore, Chen et al. (2007) observed that *F. mosseae* significantly decreased As concentrations in both plant shoots and roots, which also correlated with the fungus’s colonization. While fungus significantly increases the P supply to the alfalfa, proving that hyphal uptake of As and Chaturvedi et al. (2021) studied the Pea (*Pisum sativum*) *F. mosseae* symbiosis for phytoremediation of soil contaminated with Cd, Pb, and As. It has been found that *F. mosseae* led to an increase in plant growth, concentration of photosynthetic pigments, carbohydrates, nitrogen (N), and defense antioxidants, as well as a decrease in proline, all of which were statistically significant. Ultimately, those changes also help to protect the plant against hazardous pollutants. Furthermore, this association resulted in the removal of those pollutants from the soil to a considerable extent.

In contrast, a study conducted by Gu et al. (2017) yielded mixed results regarding the interactions between mycorrhizae and plants. Their study showed that showy stonecrop (*Hylotelephium spectabile*) and Purple Heart (*Tradescantia pallida*) survive under heavy metal-polluted soil, along with a mycorrhizal association, while exhibiting increased biomass in both roots and shoots. For example, when compared to the non-inoculated condition, stonecrop showed the most significant growth response to mycorrhizal inoculation, with increases in shoot and root biomass of 196 and 263%, respectively. The researchers measured the concentrations of Cd, Cu, Pb, and Zn in the plant’s tissues and recognized that metal concentrations in stonecrop shoots were unaffected by mycorrhiza. In contrast, Pb, Cu, Cd, and Zn concentrations in roots rose considerably by 108, 112, 34, and 19%, in comparison to the non-inoculated control. Furthermore, the study results indicated that the AMF inoculation did not affect the purple Heart plant’s shoot or root metal uptake, except for a 36 and 136% increase in Zn and Cd, respectively, in the roots. The outcomes also showed that AMF had a minimal effect on the uptake of Zn and Cu by the shoots of both plants.

#### 4.3.3 Nutrient

Endophytes solubilize micro- and macronutrients available in the soil, supporting the mobilization and uptake of these nutrients by plants. The activity of endophytic fungi is well recognized in reducing the incidence of Fe deficiency in plants. Around the roots of non-graminaceous plants, certain endophytes synthesize and excrete phytosiderophores that solubilize Fe (III) by binding to mugineic acids (MA). This conjugation results in the formation of Fe (III)-MA, eliminating the need for plants to reduce Fe (III) to Fe (II) in order to absorb rhizospheric metals (Watts et al., 2023). Those fungi are also important in N fixation, as they fix more N within the plant, which is a more favorable environment for N fixation due to the low partial oxygen pressure (Rana et al., 2020). In legume plants, endophytic fungi also promote nodulation and N fixation even under low N availability (Xie et al., 2019). According to the literature, rather than the endophyte associations, the benefits of mycorrhizae in organic N and P accumulation for plants have been well studied. When AMF interacts with plants, it increases the availability of nutrients, particularly P, and with EcM—organic N, P, and other nutrients even under hazardous environmental conditions, promoting greater plant growth (Procter et al., 2014). More precisely, mycorrhizal fungi have been found to enhance crop productivity, increasing plant height, leaf area, biomass, and seed yield, even under lower soil nutrient content (Beslemes et al., 2023). Therefore, numerous studies have been conducted to date on mycorrhizal fungi; thus, here, we primarily discuss the alterations in crop-mycorrhizal interactions, particularly those involving AMF.

In a study, Zhu et al. (2016) demonstrated the response of AMF community composition to fertilization in the rhizosphere soil of maize crops. The findings suggested various fertilization regimes had a considerable impact on AMF diversity in the maize rhizosphere. Furthermore, the addition of organic manure is the most significant factor positively influencing the composition of AMF. Notably, the input of N and P fertilizers is also recognized as the next driving factor for the AMF composition. Furthermore, testing with 2-ethylnaphthalene and 2,6,10-trimethyltetradecane showed an adverse effect on AMF—*Glomus* relative abundance; however, 3-methylbiphenyl showed a positive correlation with *Rhizophagus*. This implies that changes in fungal composition may also impact their interaction with host plants. In a separate study, Beslemes et al. (2023) investigated the contribution of AMF inoculation on the growth and productivity of two-rowed barley crops in conventional and organic cropping systems. The researchers also obtained similar results to those of Zhu et al. (2016), as the organic cropping system supports the significant influence of AMF colonization on host plants, leading to increased crop productivity. According to the findings, nutrient availability is sometimes not affected by community composition, particularly in the case of root-associated fungi. However, the effect on plant growth is also supported by the fungal associations, which supply higher nutrients to the plants than those regularly taken (Maciá-Vicente et al., 2022). It is noteworthy to highlight the results of the study conducted by Wang et al. (2022b), as their findings provide additional support for the points discussed above. Here, the researchers have tested different AMF inoculations on cherry tomato crops under varied nutritional conditions, attempting to understand their effect on AMF and host-plant interactions. In their pot experiment, Wang et al. (2022b), used three different nutritional treatments: high nutrition level (1:1, volume ratio of peat soil to sand), medium nutrition level (1:2, volume ratio of peat soil to sand), and low nutrition level (1:3, volume ratio of peat soil to sand). The four fungal inoculation treatments included *F. mosseae*, *Glomus versiforme, Rhizophagus intraradices*, and an equal mixture of all three fungi. They have recognized highly significant differences between the various AMF inoculation treatments in the root mycorrhizal infection of cherry tomatoes. Furthermore, Wang et al. (2022b), found that the amount of nutrients in the soil, the type of fungi inoculation, and their interactions all had a significant impact on the accumulation of N and P in *S. lycopersicum*. Overall, at medium and low nutrient levels, the infection rates of *G*. *versiforme* and the mixture were significantly greater than those of *F*. *mosseae* and *Rp*. *intraradices*, and at high nutrient levels, the infection rates of *G. versiforme* exceeded those of *F*. *mosseae*. Additionally, it is reported that the crop exhibits different growth performances under four fungal treatments and three nutrient levels. Wang et al. (2022a) also observed that the treatments had significant effects on flower number, fruit number, fruit biomass, and reproductive allocation of the crop. By accounting for all these results, the researchers concluded that the nutritional level directly alters the plant-AMF interaction.

#### 4.3.4 Temperature

Temperature plays a crucial role in the metabolite activities of plants, and increased temperature may have a deleterious effect on their physiological functions and growth. Climate change and the temperature rise are among the pressing issues in the agricultural sector, as evidenced by the failure of crop farming and the inability to achieve the expected harvest (Habib-Ur-Rahman et al., 2022; Janni et al., 2023). Moreover, the temperature rise alters the plant–fungal interaction both positive and negative ways, depending on the lifestyle of the plant and the fungus. When plants are associated with pathogens, the disease caused by the fungi may be more prevalent due to an increase in the virulence of the pathogenic fungi, this has been greatly presented by Waheed et al. (2023) in their review. Note that there is an optimum temperature range in each of the plant–fungal interactions at which the disease develops, for instance, 23.8°C is the optimum temperature for the White Rot disease in grape berries caused by *Coniella diplodiella* (Ji et al., 2021), while 30°C is the optimum temperature for root rot fungi *F. solani*, and 15°C is the optimum temperature for *F. tricinctum* to cause the disease in cotyledons, soybean (Yan and Nelson Jr, 2020).

In an earlier study, Park (1990) attempted to understand the infection of *Puccinia striiformis* in wheat seedlings, as well as the effects of temperature changes under both laboratory and field conditions. Based on the laboratory study, the researchers expected that no infection would occur at or above 20.8 + 0.2°C, where infection declined from 100% at 15.4°C to 0.8% at 20.5°C. However, under field conditions, the infection rate is higher between 19 and 30°C, showing difficulties in predicting which temperature range can exactly influence significant changes in plant–fungal interaction. The molecular-based answer for such changes is still lacking. Onaga et al. (2017) observed that *M. oryzae* plant fungal biomass is significantly higher at 35°C than at 28°C and also recognized the elevated level of putative fungal effector genes in plants exposed to 35°C, compared to lower temperatures. Considering all those, Onaga et al. (2017) highlighted that raising the temperature may facilitate *M. oryzae* infection by weakening plant resistance and hastening the pathogen’s colonization of plant tissues.

The adaptation of pathogens to global warming has been reported; however, the patterns and mechanisms of such adaptations in many plant pathogens remain to be understood. To fill this gap, Wu et al. (2022) tested the genotypes of *Phytophthora infestans* under five temperature regimes. They have noted that phenotypic plasticity contributes ~10 times more than heritability measured by genetic variance. Additionally, the expression of genetic variation and the relationship between local temperature and pathogen aggressiveness have been found to be altered by the experimental temperatures. The variance in aggressiveness is exacerbated by raising the experimental temperature. Pathogens from warmer climates caused less disease than those from cooler climates at low experimental temperatures, whereas the opposite was true at higher experimental temperatures. On the other hand, fungal symbionts provide more benefits to their host plants by protecting them from temperature fluctuations. Mathur et al. (2021) investigated the impact of high-temperature stress on plant physiological traits and mycorrhizal symbiosis in maize. It has been recorded that the vegetative and reproductive growth of maize, from germination to grain filling, is impacted by higher temperatures (35°C and above). The results of their experiment show that AMF interaction helps improve the plant’s photosynthesis rate by increasing N and Mg content, along with enhanced carbohydrate and sugar accumulation. The researchers further reported that AMF prevents damage to the photosynthetic apparatus (PSI and PSII) of the host plant due to the higher temperatures. Furthermore, Mathur et al. (2021) reported that enhanced photosynthesis, accompanied by improved soil quality and crop growth, leads to the mitigation of multiple malformations in the physiological characteristics of the host plant.

#### 4.3.5 Water availability

Both fungal communities and plants respond directly to the soil water regime, thereby affecting their interactions (Erlandson et al., 2016; Xiao et al., 2022; Chen et al., 2023). On one hand, plants generally increase their susceptibility to phytopathogens due to drought stress. On the other hand, they also increase their resistance against those phytopathogens. The morphological characteristics of the plant root are one of the factors that determine its relationship with fungi. It has been recognized that plants with thin root systems exhibit lower dependence on AMFs for water uptake, due to their ability to acquire water efficiently (Lozano et al., 2021). Most EcMFs typically form rhizomorphs and hydrophobic mycelia, which help plants mitigate the impact of drought (Castaño et al., 2023).

Boczoń et al. (2021) described in detail how the plant–fungal interactions change under water scarcity (drought) due to climate change. According to them, drought reduced the overall prominence of mycorrhizal associations with the plants, leading to reduced further water uptake and alterations in metabolic pathways due to ATP and nutritional deficiencies. Next, impairment of plant tissues and chlorosis of the leaves occur, leading to a switch in the fungus’s endophytic lifestyle to pathogenic, causing disease in the hosts. In addition, Boczoń et al. (2021) noted that these changes weaken the plant’s defensive system, even against pests, and make it more susceptible to invertebrate attacks.

Joachin et al. (2023) studied how water availability influences the effective specialization of a fungal pathogen in its interactions with plants. Under low, average, and high-water treatments, they established paired congeners of three native and three non-native species of coastal prairie plants, with or without the pathogenic soil fungus *Fusarium incarnatum-equiseti* species complex 6b. The *Fusarium* treatment demonstrated greater adverse and species-specific effects on plant biomass at high water availability than at low water availability for all examined plant species. Furthermore, the results of Joachin et al. (2023) supported the discussions by Benítez et al. (2013). They have emphasized that the effective specialization of pathogens promotes the coexistence of plant species, for instance, via negative plant–soil feedback or the Janzen-Connell hypothesis (Comita et al., 2014). Under water scarcity, endophytic and mycorrhizal fungi show various alterations to host plants, depending on the species and the combination of other environmental factors (Dastogeer, 2018; Ahlawat et al., 2022; Frew, 2023). For instance, drought conditions may lead to the production of various plant biochemical compounds, also with the help of fungi, which mitigates water stress conditions (Boczoń et al., 2021). Hubbard et al. (2014) demonstrated that endophytic Ascomycetous fungi enhance the heat and drought tolerance of wheat (*Triticum turgidum*) in terms of grain yield and second-generation seed viability. Later, in a more comprehensive study, Miranda et al. (2023) found that the endophytic fungus *Zopfiella erostrate* substantially colonizes wheat and tomato roots, contributing to enhanced plant nutrient mineralization and water uptake under water deficiency. Their study showed the significant effect of the fungus on plant growth; however, the researchers did not observe any considerable changes in fungal root colonization under water-deficient conditions compared with the well-watered plants. Nevertheless, the fungus exhibited higher photosynthetic efficiency, decreased enzymatic activity (catalase and ascorbate peroxidase), altered glutathione reductase activity, lower hydrogen peroxide concentrations, and a significant drop in lipid peroxide accumulation. The studies indicated that such a change or activation of antioxidant compounds and enzymatic activities worked against oxidative damage generated by water-deficient conditions (Piri et al., 2019; Kaur and Saxena, 2023).

In an experiment with *F. mosseae* on tomato, Bitterlich et al. (2018) emphasized the importance of AMF in alleviating the drought impact on the plants. They recognized the considerable amount of available water content reserved by *F*. *mosseae*. To a great extent, fungal restoration of plant hydraulic status and increased plant transpiration were also observed with the decline in soil water content. In addition to water scarcity, soil waterlogging conditions also influence plant–fungal interactions. According to the study by Calvo-Polanco et al. (2014), the fungus *R. irregularis* benefits tomato plants under flooded conditions. They have gained a molecular-level understanding, as evidenced by the increase in root hydraulic conductivity due to the fungus, which is related to the upregulation of aquaporin gene expression, specifically *GintAQP1* and *SlPIP1*;7. Further studies have demonstrated that mycorrhizal fungi stimulate the production of 1-aminocyclopropane-1-carboxylic acid (ACC) and ethylene (Et) in plants, thereby enhancing their resistance to flooded conditions (Cruz et al., 2000).

#### 4.3.6 CO_2_ concentration

The rise of atmospheric CO_2_ has been observed over recent decades, which is linked to climatic changes. Elevated CO_2_ (eCO_2_) is beneficial for crops, as it increases the efficiency of photosynthesis and growth; however, the overall impact is not entirely understood. Furthermore, the interaction between plants and fungi is also affected by changes in CO_2_ (Smith and Luna, 2023).

Chakraborty and Datta (2003) evaluated the aggressiveness of *Colletotrichum gloeosporioides* in shrubby stylo (*Stylosanthes scabra*) that causes anthracnose disease. Considering their 22-year field experiment period, Chakraborty and Datta (2003) found a fourfold difference in disease severity levels between isolates with the lowest and highest disease severity levels. Prior to 1987, both weakly (severity < 0.6) and moderately aggressive (severity 0.6–0.8) isolates were found; however, between 1991 and 1999, the distribution became more uniform, with aggressive isolates (severity 0.8–1.2) and highly aggressive isolates (severity > 1.2) towards the host plant. They have recognized that these changes mainly happen due to the eCO_2_. Further, they have correlated this scenario with the controlled environment study. The experiment demonstrates that increasing CO_2_ levels (from 400 ppm to 700 ppm) enhances the pathogen’s aggressiveness. In their experiment, Karnosky et al. (2002) observed that eCO_2_ (560 ppm, 200 ppm)/O_3_ (1.5x ambient) occurrence and severity of leaf rust caused by *Melampsora medusae* on trembling aspen (*Populus tremuloides*). They have found that seasonal exposures to O_3_ and CO_2,_ as well as O_3_ alone, both resulted in significantly increased rust occurrence and severity, three to five times. Leaf surface topography, microroughness, and physicochemical characteristics (e.g., chemical composition and epicuticular wax structure) collectively determine leaf surface properties, such as wettability, and this is recognized as a key consideration for changes in host-fungus interactions. The wettability of the leaf surface significantly increases the incidence of *M. medusae* by positively influencing the fungal attachment and further infection process. In the contest, some studies reported enhanced disease resistance under eCO_2_. For instance, Hibberd et al. (1996) and Mikkelsen et al. (2015) found that eCO_2_ (700 ppm) shows resistance against the *Erysiphe graminis* and *Blumeria graminis,* respectively, associated with barley. Williams et al. (2018) recognized the increased resistance of *Arabidopsis thaliana* against necrotrophic *Plectosphaerella cucumerina* infection under the eCO_2_ (1,200 ppm). Furthermore, the study by Williams et al. (2018) added value to Karnosky et al. (2002), as it considered in-depth biochemical mechanisms and molecular bases to explain disease resistance. Of this, Williams et al. (2018) noted a 69.3 and 69.4% increase in SA and JA accumulation under eCO_2_, respectively. They have further identified that eCO_2_ alters basal and SA-induced *PR1* gene expression, which is responsible for stress resistance. In line with this, Mhamdi and Noctor (2016) also recognized increased disease resistance in *A. thaliana* (along with other plants, such as *Phaseolus vulgaris*, or beans) against *B. cinerea* under 3,000 ppm of CO_2_. They have also highlighted that eCO_2_ triggers SA accumulation and is partially linked to metabolic effects involving redox signaling. This is supported by the fact that complete priming of the SA pathway and the corresponding resistance to high CO_2_ are prevented by genetic modification of redox components, including glutathione levels and NADPH-generating enzymes. Furthermore, they have emphasized that this is also true for other crop plants studied, such as beans. In addition, working with *Cochliobolus miyabeanus* that infects rice, Dorneles et al. (2020) recognized (under 700 ppm of CO_2_) higher activity of the enzyme’s ascorbate peroxidase, catalase, chitinase, peroxidase, polyphenoloxidase, superoxide dismutase, enhanced phenolic compounds, and lignin concentration, indicating defense responses.

The interaction between plants and endophytic fungi may vary in response to eCO_2_. According to their experiment, Christian et al. (2021) found that a community of endophytic fungi declined with eCO_2_, which is opposite to the findings of Alberton et al. (2010). Liu et al. (2023) investigated the diversity and colonization of eCO_2_ and AMF. They have recognized that with eCO_2_, the diversity of AMF increases, while decreasing root colonization with host plants, such as maize and wheat.

### 4.4 Synergistic effects create more benefits for plant–fungal interactions

The combined application, or co-inoculation, offers a nuanced understanding of plant–fungal interactions, although it can sometimes be extremely challenging to predict the outcomes (Niu et al., 2021). However, several studies have assessed the co-inoculation of different groups of non-pathogenic fungi and found that it has more beneficial effects on plants and crops. In a foremost study, Khaekhum et al. (2021a) tested the growth and yields of sunchoke with co-inoculation of endophytic fungi *Exserohilum rostratum* NMS1.5 and an AMF *Glomus etunicatum* UDCN52867 g.5. They found that such application gives significant growth improvement (some parameters), and tuber yield of sunchoke, compared to the application of chemical fertilizers. Furthermore, their results demonstrate superior outcomes compared to those previously reported by Khaekhum et al. (2021b) and Nacoon et al. (2021), indicating a synergistic effect of endophytic fungi and AMF. Co-inoculation is also beneficial to plants, as it helps mitigate the aforementioned biotic and abiotic stresses. In an early study, Leeman et al. (1996) demonstrated the suppression of Fusarium wilt disease in radish by co-inoculation of *Pseudomonas* spp. and root-colonizing fungi, including *Acremonium rutilum*, *F. oxysporum*, and *Verticillium lecani*. They have found that *F. oxysporum* and *V. lecanii* induced systemic disease resistance, hence suppressing the disease. Nevertheless, when co-inoculation of *A. rutilum, F. oxysporum*, or *V. lecanii* with *Pseudomonas* spp. strains WCS358, WCS374, or WCS417, or their pseudobactin-minus mutants, showed a significant suppression of the disease compared to the control treatment.

Seneviratne et al. (2015) evaluated the single and combined application of a gram-negative bacterium and a fungus (*Aspergillus* sp.) on *Z. mays*, protecting the plants from growth inhibition. Plants treated with combined microbes increased their uptake of Ni by 13%, Mn by 52%, and Cr by 83%, compared with non-inoculated plants. Further combined application shows a higher heavy metal accumulation factor in plants compared to the single application. For example, the Mn plant accumulation factor is 0.26 ± (0.02) for control, 0.14 ± 0.03 for fungi, 0.18ab ± 0.07 for bacteria, and 0.23b ± 0.05 for combined application. However, the transfer factor (TF) was reported to be lowest in the combined treatment (for Mn, control-0.20 ± 0.05, fungi-0.13a ± 0.03, bacteria-0.11a ± 0.02, combined-0.10a ± 0.04). They further concluded that the combined application resulted in a reduction of heavy metal accumulation in plant shoots, thereby rendering the grains harmless for consumption. While Zou et al. (2024) recorded pronounced heavy metal tolerance (Cd) in *Robinia pseudoacacia* under an excess N condition due to the co-inoculation of *Mesorhizobium huakuii* and *F. mosseae*, compared to the individual application, their study also reported that, although heavy metal accumulation is greater in roots after microbial inoculation, accumulation in shoots was reduced. Furthermore, it demonstrates that such applications enable plants to resist or repair damage caused by Cd effectively. Ważny et al. (2018) demonstrated the importance of the combined application of AMF, *Rhizoglomus intraradices*, and endophytic fungi, *Mucor* sp. or *T. asperellum*, over single inoculation in mitigating the effects of heavy metals (Zn, Cd, Pb) on *Lactuca serriola*. Other than that, they have concluded that the combined application of these fungi could also support the plants in withstanding drought conditions.

Gasemi et al. (2023) studied the beneficial interaction of the combined application of plant growth-promoting rhizobacteria (*Azotobacter, Bacillus lentus*, *Pseudomonas genus,* and *Pseudomonas putida*) and AMF (*G. etunicatum*, *Rp. intraradices*, and *G. mossea*) with *Dracocephalum kotschyi*. The findings suggested that co-inoculation of those fungi creates more positive effects on the host plant than independent applications, such as an increase in plant height, leaf number, yield, essential oil content, chlorophyll content, carotenoid concentration, relative water content, total soluble sugars, and proline content. The most important consideration of their study is that those co-inoculated microbes deliver the aforesaid benefits to the host plants under water deficit stress conditions. In a similar study, Barzegari Barogh et al. (2023) co-inoculated *Azotobacter chroococcum* and *Pseudomonas putida* with AMF (*F. mosseae*, and *Rp. intraradices*) on potato plants. In addition to the beneficial effects reported by Gasemi et al. (2023), Barzegari Barogh et al. (2023) highlighted the importance of improving nutrient content. For example, compared with the control, the interaction of *P. putida* at 100 mL and *Rp. intraradices* increase the potato minituber number (116%), minituber weight (181%), shoot dry weight (248%), root dry weight (120%), chlorophyll content (57%), carotenoid content (10%), ascorbic acid (8%), proline (18%), total soluble solids (TSS, 49%), TSS to titration acidity (46%), phosphorus (72%), potassium (27%), zinc (24%), and Fe (17%). Zai et al. (2021) tested two fungi species, AMF- *F. mosseae*, and a phosphate-solubilizing fungus, *Apophysomyces spartima,* with Beach plum (*Prunus maritima*) host to understand the helpfulness of interaction in nutrient uptake and photosynthesis under a salt stress environment. The findings are also significant for nutrient uptake (P and N), and higher values for photosynthesis. Similarly, the study by Lee et al. (2015) demonstrates the alleviation of salt stress and improved maize growth through the combined application of *G. etunicatum* and *Methylobacterium oryzae* CBMB20.

### 4.5 Challenges and required advancement

#### 4.5.1 Multiomics approaches

The study of multiomic techniques, including the integration of genome, metagenomics, metabolomics, volatilomics, and spectranomics data, in plant–fungal interactions appears to be scarce. However, such an application will provide an in-depth understanding of the biology and physiology of both the plant and the fungus when they are associated (Shakeel et al., 2022). These multiomic strategies can unravel the functional roles of different fungal species within agricultural ecosystems, improve our understanding of how co-infecting fungi may synergistically modulate the plant’s defense mechanisms, often leading to enhanced pathogenicity and altered nutrient acquisition strategies. Analyzing the microbial community dynamics beneath the plant surface through this methodology sheds light on how co-infection scenarios can lead to unique fungal interactions that ultimately impact plant productivity and health, including the identification of microbial modulation of key plants’ genetic potentials and metabolic pathways (Hori et al., 2021; Doni et al., 2022; Luo et al., 2022). Also, multiomics can facilitate the discovery of novel bioactive compounds produced during interaction, further expanding the toolkit for sustainable agriculture (Kumar et al., 2023). For instance, genomics studies can identify genes in microbes that are responsible for nutrient acquisition or phytohormone production, which in turn enhance plant growth (Wójcik et al., 2023). Transcriptomics investigations reveal how plants respond to microbes at the gene expression level, highlighting signaling pathways activated during symbiosis or pathogen defense (Van Dillewijn, 2008; Mukherjee, 2022). There are several limitations to using multiomic approaches. Such as in gene expression, replication of environmental conditions, complexity of microbial communities, and difficulties in obtaining sufficient materials (e.g., Rhizosphere fungi), its wide use is restricted (Chiquito-Contreras et al., 2024). Overall, in multitopic approaches, several limitations exist, including gaps in taxonomic or chemical compound databases, which can make comparison and verification challenging. Instead, higher costs for sample analysis, advanced technologies with specific infrastructure facilities, and a lack of expertise are the main considerations, particularly in developing countries. Those factors further restricted the application of multiomic techniques (Crandall et al., 2020).

#### 4.5.2 Network mapping

Network mappings of plant–fungal interactions are crucial for understanding how microbes influence plant metabolism, stress responses, and immune systems (He et al., 2021). Of this, genome-wide association studies (GWAS) have been a recent focus; for example, Bergelson et al. (2019) identified a few significant Quantitative Trait Loci (QTLs) associated with *Arabidopsis thaliana* root microbial species richness and community structure. These QTLs are implicated in plant immunity, cell wall integrity, and the development of roots and root hairs. A study by He et al. (2021) developed a behavioral ecology model to define the strengths of mutualism, antagonism, aggression, and altruism between each pair of root microbes of *A. thaliana*. It showed the importance of such studies in selecting microbes that benefit sustainable plant growth. De Vries et al. (2018) indicated that the inherent mechanisms of microbial relationships in response to environmental disturbances can be uncovered by examining the properties of co-occurrence networks. Agler et al. (2016) noted that recognizing network hubs and their significance in microbial community structure has important implications for understanding interactions between microbes and can aid in the development of targeted biocontrol strategies in the future.

#### 4.5.3 Influence of indigenous fungi

One of the overlooked aspects of plant–fungal interactions is the impact of indigenous fungi on plant growth and protection. Studying native fungi is limited, which could be due to their lower infection potential, and they may not be able to get sufficient inoculum that non-native fungi offer (Deguchi et al., 2021; Wu et al., 2021b). However, several studies have revealed that native fungi also have significant potential to enhance crop productivity in various dimensions, making their interaction with plants a crucial consideration (Koziol and Bever, 2023). One such example is the communication of plants through fungal mycelial networks. This is widely discussed in relation to mycorrhizal fungi; the fungal mycelia allow signals to be sent between plants. Recent research has demonstrated that these mycorrhizal networks serve as an information highway, facilitating the exchange of defense signals, allelochemicals, and nutritional resources (Luo et al., 2023). Nevertheless, this aspect has not been extensively discussed in the agricultural context, although it has been explored in other ecosystems (Gorzelak et al., 2015). On the one hand, this could be due to the shift in paradigm towards intensive, input-driven practices that prioritize high-yield crop production. Synthetic fertilizers and pesticides became the standard tools for increasing productivity, as they provided immediate and visible benefits to plant growth. However, these activities may alter soil properties and accumulate pollutants, which could negatively impact indigenous or native fungi, thereby hindering their potential benefits (Semenov et al., 2022; Steiner et al., 2024). On the other hand, the intricacy of biological interactions is not attracting researchers, as it is a challenging task (Aleklett et al., 2021).

#### 4.5.4 Commercial fungal formulations

It has long been recognized that the importance of fungal biocontrol agents is higher among academic communities (Wallis and Sisterson, 2024). In contrast, the commercial application is still not widely adopted worldwide due to its limitations, including a short shelf life, reduced efficacy, commercialization issues, and legislative procedures (Bamisile et al., 2021; Palmieri et al., 2022). Production quality control is one of the crucial steps in fungi formulations. The quality control process initiates with evaluating the inoculants, microbial strains, carriers, and the final product, as well as ensuring proper labeling and commercialization. To obtain legal acceptance, it is essential to properly identify fungi species or strains prior to mass cultivation. Furthermore, the cultures must be sterile, with high viability and germination capacity. Regulation regimes in the European Union and India specifically concern minimum viable spores and acceptable variation in microorganism concentrations (Ghorui et al., 2025).

When discussing commercial formulations, it has been recognized that the importance of developed fungal strains extends beyond indigenous fungi. In the current complex agricultural system, indigenous fungi may not be the most competitive option; however, it is also essential to identify more effective species for future formulations (Gohel et al., 2022; Díaz- Urbano et al., 2023; Erdoğan and Sağlan, 2023). Keep in mind that one of the foremost things to discuss in the fungal formulations is the viability of fungi. It is typically expected to be effective for at least 6 months and preferably for about 2 years. The formulation could be liquid-based or solid-based; however, both forms have their advantages and disadvantages. The liquid media could be easily controlled and supplemented with nutrients, although it required complex fermentation equipment. The solid-based formulations are relatively simple; in contrast, it is a challenge to supply the necessary nutrients to the fungi, and maintaining the viability of the fungal inoculum under low water content in the formulations can be complex (Teixidó et al., 2022). The researchers also made significant efforts to enhance the packaging and shelf life of fungal formulations. For example, Jeong et al. (2022) utilized modified atmosphere packaging (MAP) to prolong the shelf life of *Metarhizium anisopliae* conidia. They have found that MAP with 30% CO_2_ + 70% N_2_ retains 80.5% conidial viability after 28 days. However, the challenge here is the storage condition, as achieving higher shelf life with higher conidial viability requires cold storage facilities. Jeong et al. (2022) reported that MAP-treated conidia have a longer half-life when stored at 4°C compared to 25°C. In an independent study, Silva et al. (2024) attempted to extend the shelf life of a rice flour-granular formulation based on *Trichoderma* conidial biopesticide. Similar to the result of Jeong et al. (2022), Silva et al. (2024) evidenced that cold storage of the formulation is necessary for a longer shelf life (up to 24 months), which may not always be convenient for sellers or farmers in practice, particularly in developing countries.

Scientific evidence also indicated that the demerits of such applications—could be highly specialized against the targeted disease or the pathogen, and the controlling ability is highly dependent on the various biotic (e.g., host plant, metabolites and enzymes synthesized by host plant, and natural microflora of the host plant and their interaction) and abiotic (e.g., microelements, nutrients, pH, temperature, ultraviolet (UV) radiation, and water activity) conditions (Bonaterra et al., 2012). Therefore, the prediction of the effectiveness when applied to the field could be reduced, inconsistent, and dependent (Boro et al., 2022). For example, the study by Wei et al. (2019) can provide insight; the researchers tested the endophytic fungus *Fusarium solani* against *Verticillium dahliae* in Cotton plants. They have found that the biocontrol potential of the fungi is higher in efficacy in the greenhouse experiment. In contrast, when tested in the field, the efficacy decreased (Table 1). Nevertheless, numerous studies have shown that fungi biocontrol agents can be effective against phytopathogens; however, these findings are limited to greenhouse experiments (Herrera et al., 2020; Catambacan and Cumagun, 2021; Safari Motlagh et al., 2022).

## 5 Prospects

Along with the advancement of technology, a significant understanding of plant-fungal interactions has been gained today. The mechanisms behind phytopathogenic fungal infection, host immunity, and disease susceptibility have been extensively studied at the molecular level. However, due to the complex nature of those associations, considerable studies still need to be conducted. For instance, studies on plant–fungal interactions under co-infection and co-inoculation (synergistic) of beneficial fungi are limited and have not revealed the proper association.

Comparing plant–fungal interactions between natural and agricultural systems is important for future research. Such comparisons can help identify general mechanisms of the interaction that cope with environmental stresses, especially with the aid of modern omics technologies. Additionally, there is a potential for obtaining fungal strains that are beneficial for crop health. Understanding and harnessing the interactions between plants and fungi can significantly improve agricultural practices. Nevertheless, understanding the conditions that need to be overcome and carefully selecting the type of symbiotic fungi group is vital to achieving the best results.

Furthermore, future studies should prioritize identifying new symbiotic relationships, unraveling the mechanisms of plant interaction, and exploring the various effects of these relationships on plants. Additionally, the use of indigenous fungi to enhance agricultural productivity and crop protection through their interaction with host plants could be a promising area for future research. In the context of disease control, a critical but often overlooked consideration is the prevention of virus infections in plants. However, a few studies have highlighted the potential of plant–fungal interactions in controlling these viruses, underscoring the importance of this issue and the need for further research.

## 6 Conclusion

The pathogenic association between a fungus and a plant is one of the most recognized phenomena in nature. The use of the Scopus database only to extract data could hinder the precise development of the field, as many publications are not included in this database. Nevertheless, the platform is also vital for extracting the most acknowledged studies in the field, as well as for gaining an overall picture of the field. According to the bibliometric analysis, it is evident that studies on the interaction between plants and fungi have increased steadily over the last three decades. Notably, China has emerged as a leading contributor to the field of study, accompanied by a dynamic cohort of early-career and emerging authors/researchers, a pattern that mirrors the global shift toward “green” agricultural paradigms. Yet, despite this momentum, the evidence base remains uneven across climatic zones and production systems, underscoring the need to expand inquiry across tropical and temperate agriculture contexts and to prioritize research on the world’s top-ranked major crops and high-value crops to maximize impact. On the one hand, plant–pathogen interactions continue to impose significant yield and quality losses due to the phytopathogens, reinforcing the need for improved surveillance, resistance breeding, and integrated disease management. On the other hand, mutualistic partnerships—particularly with mycorrhizal fungi and endophytes—offer compelling avenues to enhance plant growth and development, increase efficiency of water and nutrient acquisition, modulate host immunity, and confer tolerance to abiotic stressors such as drought, salinity, and heat, thus playing a significant role in climate-resilience. Strategically fostering these beneficial symbioses can reduce reliance on synthetic fertilizers and pesticides, with attendant co-benefits for soil health, biodiversity, and farm profitability. Realizing this potential, however, requires extended field-based experimental designs, standardized metadata and reporting, and integrative approaches that connect molecular mechanisms. Although it is essential to conduct advanced research (e.g., multi-omics studies), this has not been practiced adequately, particularly in developing countries, due to limited technological and financial resources; thus, the need for such involvement is pressing. Collectively, our mapping and synthesis highlight both the opportunities and constraints of plant–fungal interactions, advancing a research agenda that can translate fundamental insights into scalable, environmentally responsible strategies to safeguard productivity while reducing external inputs.

## Data Availability

The data used for bibliometric analysis can be obtained by writing to the corresponding author.

## Footnotes

^1^ https://www.scopus.com/pages/home#basic
