# Supplementary material for: Mapping of plant–fungal interactions on agriculture perception: a bibliometric analysis and systematic review
Source: Front Microbiol. 2025 Oct 10;16:1612428. doi: 10.3389/fmicb.2025.1612428 (PMC12551605; doi:10.3389/fmicb.2025.1612428)
Supplement: Supplementary file 1 [file Table_1.docx]

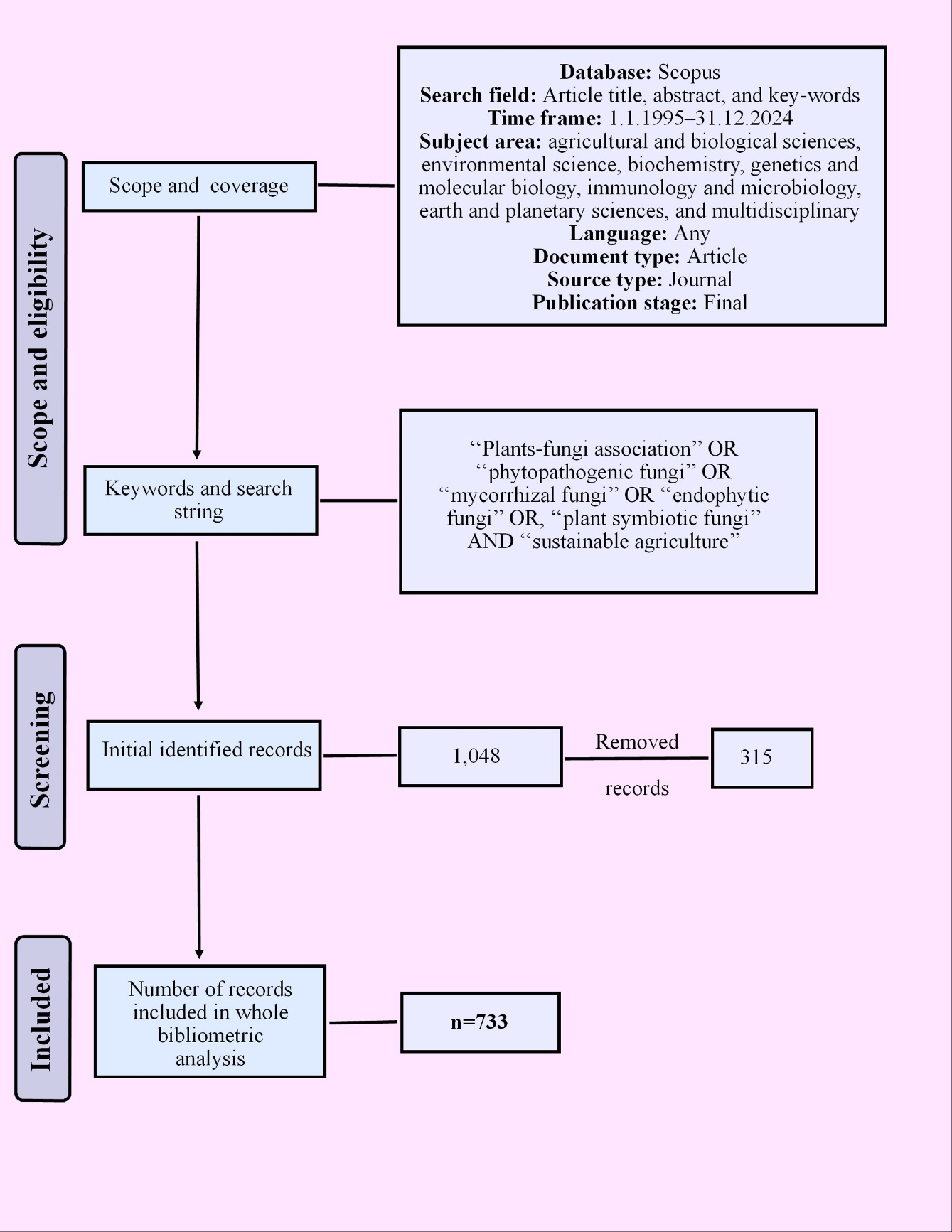


**Sup. Figure 1.** Data screening procedure through the Scopus database.


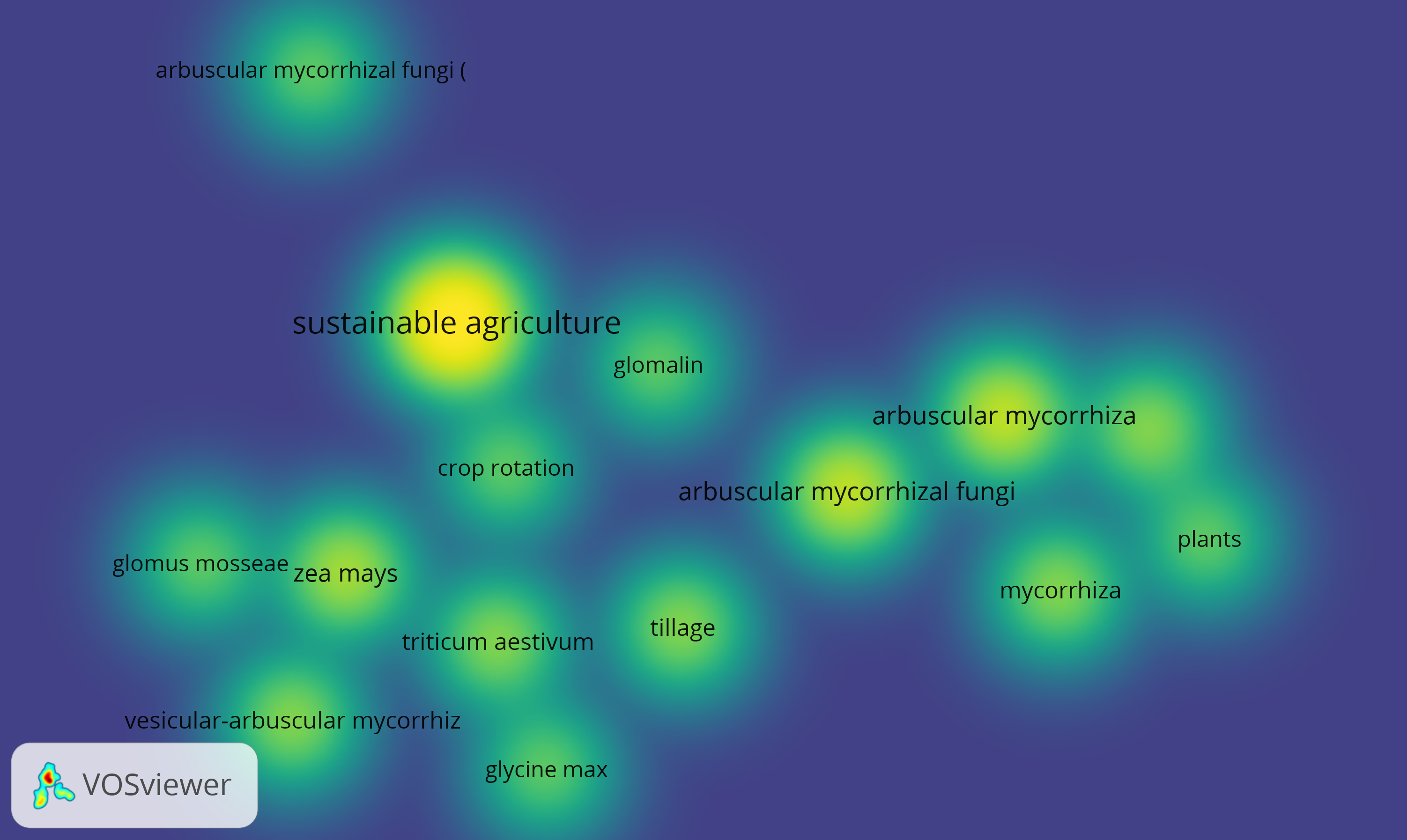

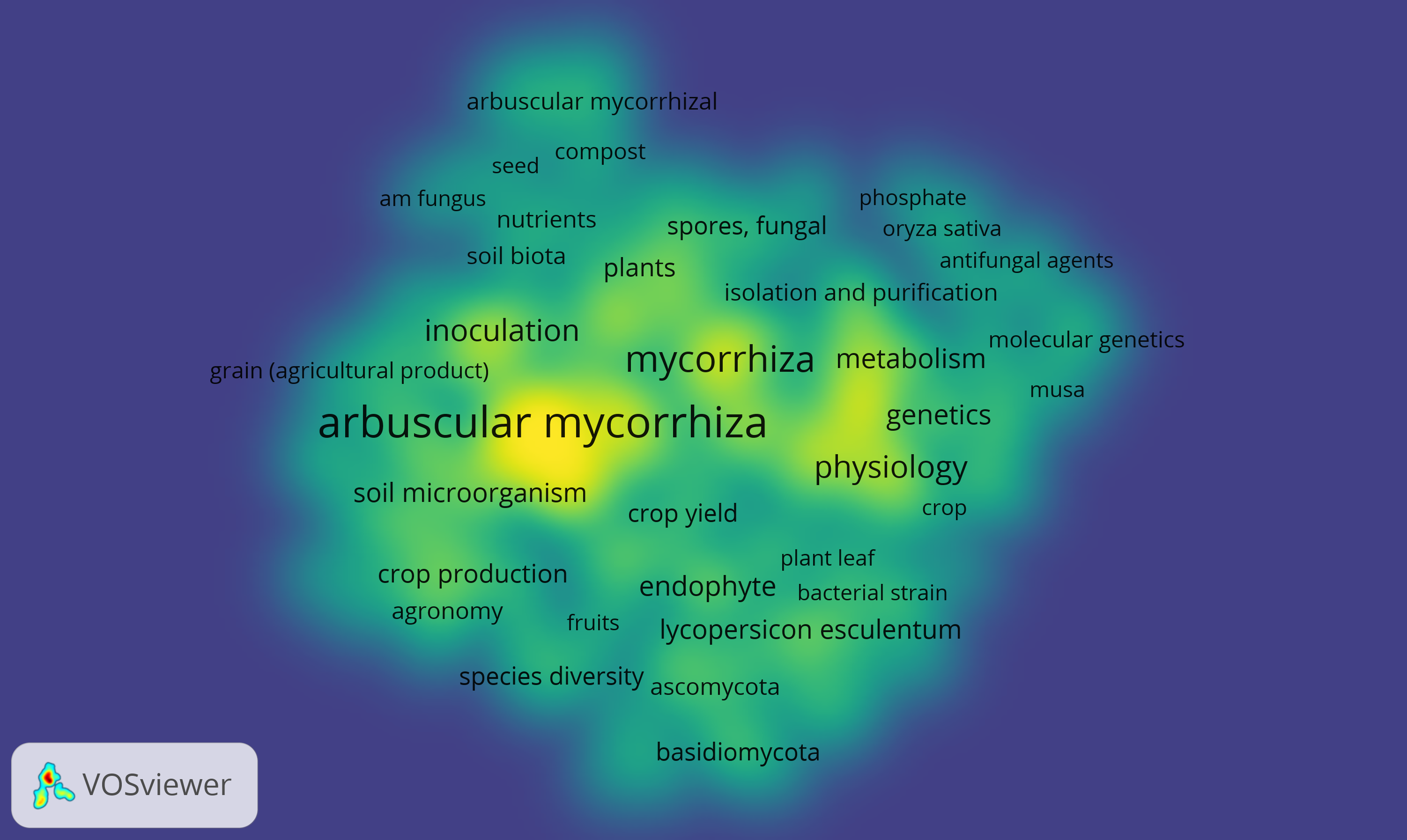

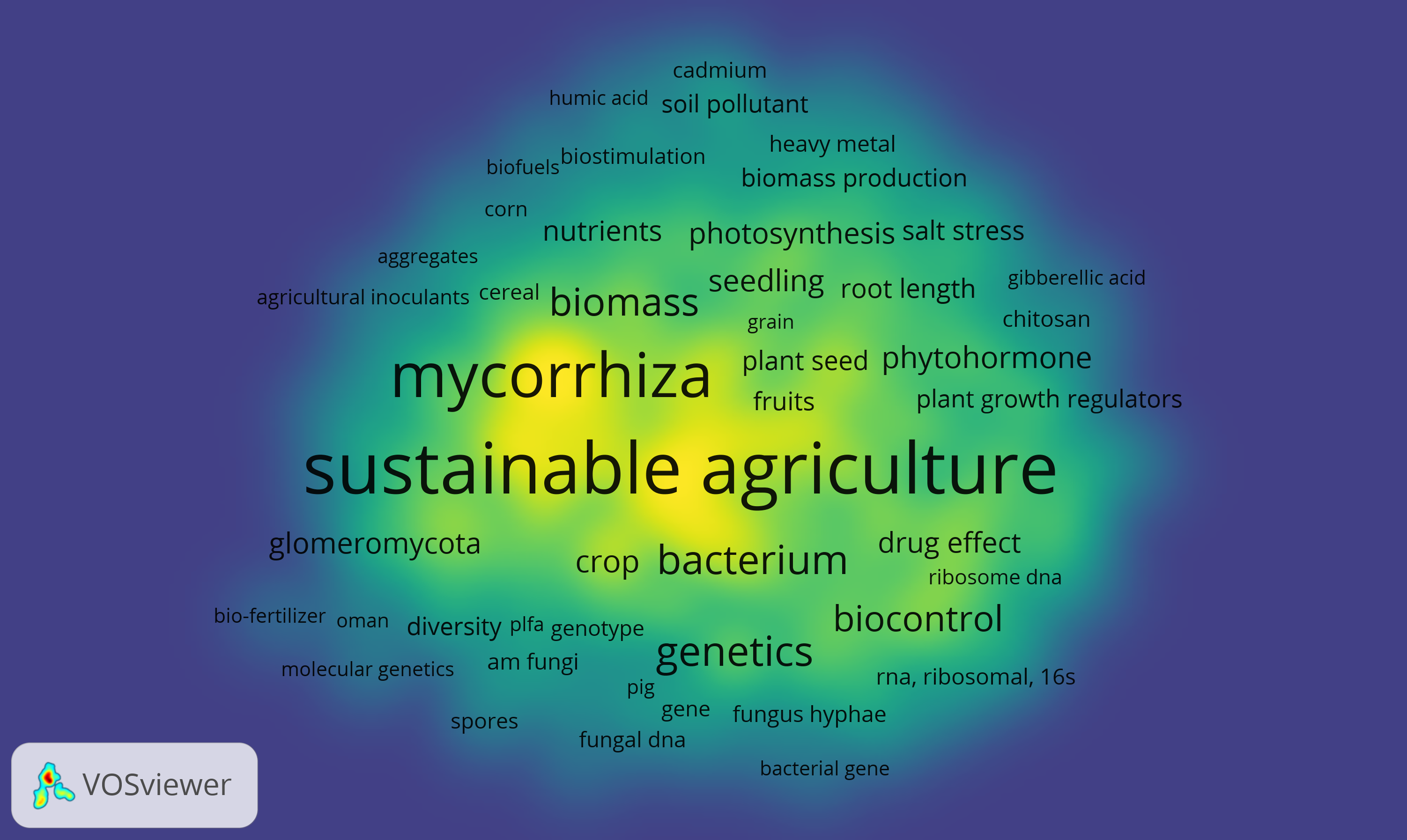


(a)

(b)

(c)

**Sup. Figure 2.** Density visualization of keywords in different phases (a). 1995–2004: 15 keywords were recorded in four clusters, with 56 links and 93 TLS. (b). 2005–2014: 180 keywords recorded in six clusters, with 4931 links, totaling 7523. (c). 2005–2014: 869 keywords were recorded in eight clusters, with 56,834 links and 90,992 TLS. The minimum number of keyword occurrences is set to three for better clarity.
